# High-resolution mapping of residential wood burning heat sources using Energy Performance Certificates: A case study of England and Wales

**DOI:** 10.1016/j.envint.2025.109537

**Published:** 2025-06-03

**Authors:** Laura Horsfall, Calum Kennedy, Nicola Shelton, Jonathon Taylor, John R. Hurst, Matthew Loxham, Adam Dennett, Pia Hardelid

**Affiliations:** a Institute of Health Informatics, University College London, London, NW1 2DA, United Kingdom; b Department of Epidemiology and Public Health, University College London, London, WC1E 7HB, United Kingdom; c Faculty of Built Environment, Tampere University, Korkeakoulunkatu 7, Tampere, 33720, Finland; d UCL Respiratory, University College London, London, NW3 2PF, United Kingdom; e Institute for Life Sciences, University of Southampton, Southampton, SO17 1BJ, United Kingdom; f Bartlett Centre for Advanced Spatial Analytics, University College London, London, W1T 4TJ, United Kingdom; g Great Ormond Institute of Child Health, University College London, London, WC1N 1EH, United Kingdom

**Keywords:** Air pollution, Spatial statistics, Data science, Built environment, Exposure assessment

## Abstract

**Background:**

Particulate matter emissions from residential wood burning are rising in many countries. Long-term exposure to fine particulate matter is strongly linked with adverse health effects including cardiovascular and respiratory disease. Policymakers and scientists need accurate tools to identify residential wood burning hotspots. However, current methods rely on spatially-misaligned, out-of-date data sources, reducing their practical utility and portability to other contexts. Furthermore, the socio-economic characteristics of residential wood burning in high income countries are poorly understood.

**Methods:**

We used open data from 26 million Energy Performance Certificates (EPCs) for properties in England and Wales from 2009–2025 to map the concentration and prevalence of wood burners within small areas. We evaluated our method against the UK national wood burning emissions inventory using national air pollution monitoring networks. We used novel open data linkages to characterise associations between area-level prevalence of wood burners and socio-economic factors including deprivation, ethnicity, and age.

**Findings:**

We identified substantial spatial heterogeneity in the concentration of wood burners, with the highest concentrations in affluent urban areas. Our concentration metric was more strongly correlated with peaks in winter PM2.5 at urban monitoring sites than estimates from the UK national emissions inventory. Prevalence of wood burners was positively correlated with age and negatively correlated with measures of social deprivation. Prevalence of wood burners in EPCs has increased since 2009.

**Conclusions:**

EPCs are a valuable data source which policymakers can use to target local interventions or extend existing restrictions on solid fuel burning. Our method is transparent, up-to-date, and portable to other countries where similar EPC data is available. The relationship between social deprivation and prevalence of wood burning heat sources highlights important issues of environmental justice. Epidemiological analyses of wood smoke exposures and health should carefully account for the confounding effects of age, deprivation, and ethnicity.

## 1 Introduction

Burning solid fuels such as coal, wood and other biomass releases harmful pollutants including carbon monoxide, nitrogen dioxide, and small particulate matter (PM_2.5_). These pollutants can cause immediate health effects and increase the risk of chronic conditions including lung and cardiovascular disease (Mehta et al., 2023; Lee et al., 2020). The World Health Organisation estimates solid fuel emissions were responsible for 3.2 million premature deaths in 2020 (World Health Organisation, 2024).

In recent decades, high-income countries have focused on reducing particulate emissions from vehicles and industry, with many regions showing dramatic improvements since the 1960s (Butt et al., 2017). However, with efforts concentrated in these sectors, residential emissions from solid fuel – mainly wood fuel – increased in many countries. Consumption of solid biomass in the European Union (EU) has doubled since 1990 (European Commission, 2024). Most of the increase has occurred since 2002, when the EU’s first renewable energy policy promoted biomass as a renewable fuel (European Commission, 2024; Booth, 2022).

Current trends in residential wood burning are concerning due to the potential health impacts of wood smoke exposures. A growing literature highlights possible relationships between wood smoke exposure and adverse health outcomes including lung cancer (Guercio et al., 2022, 2021; Piper et al., 2024). Residential wood burning has also been linked to carbon emissions and biodiversity loss (Savolahti et al., 2019; Searchinger et al., 2018).

To explore the health and climate impacts of residential wood burning at scale, scientists need to understand where wood burning is most common. High-resolution mapping of residential wood burning can also inform targeted public health interventions or awareness campaigns focused on wood burning. Targeted interventions can be effective. In the Tasmanian city of Launceton, a series of local policy measures during the 1990s and early 2000s including educational campaigns and a wood heater replacement programme reduced the household prevalence of wood stoves from 66% to 30% (Johnston et al., 2013).

However, high-resolution mapping of residential wood burning using existing data sources is challenging. National air quality monitoring networks are typically biased towards traffic pollution from major roads. Residential wood burning emissions inventories generally integrate multiple data sources at different spatio-temporal scales (Karvosenoja, 2008; Lopez-Aparicio et al., 2018; Borchers-Arriagada et al., 2024; US Environmental Protection Agency, 2023; Zalzal et al., 2024; Tsagatakis et al., 2024). Combining these misaligned data sources necessitates modelling assumptions, which can create biases in the high-resolution inventory (Vedrenne et al., 2016).

Existing residential wood burning maps are also generally uninformative about the socio-economic context of the areas where wood burning is prevalent. More broadly, despite an extensive literature characterising solid fuel usage in developing countries (McLean et al., 2019; Qiu et al., 2022), there is relatively little evidence on the socio-economic distribution of residential wood burning in high-income countries. Such contextual information could play an important role in epidemiological studies and for targeting public health interventions to specific local area characteristics.

The United Kingdom (UK) is an interesting case study for the resurgence of residential wood burning. Following the Great London Smog of 1952, where a combination of residential coal emissions and unusual weather conditions caused an estimated 12,000 deaths, the government established The Clean Air Act. The Clean Air Act included designated Smoke Control Areas (SCAs) in towns and cities. Only smokeless fuels (e.g., anthracite or gas) were permitted for heating homes in SCAs, making the UK the first country to implement legal restrictions on residential emissions.

Today, most wood burning appliances sold in the UK are SCA-compliant. However, residential wood burning remains a major contributor to ambient air pollution, comprising 11% of total PM_2.5_ emissions in 2022 compared to 4% from vehicle exhausts (Department for Environment, Food and Rural Affairs, 2025). A recent survey found that residential wood burning is more prevalent in affluent households, raising issues of environmental justice (Department for Environment, Food and Rural Affairs, 2024b). In the UK, local governments are responsible for local policies (such as setting SCA boundaries) and awareness campaigns.

Energy Performance Certificates (EPCs) are a potentially valuable data source for high-resolution mapping of wood burning heat sources. EPCs provide information on property-level characteristics including primary and secondary heat source. EPCs can be easily linked to other high-resolution open data sources including local socio-economic characteristics. EPC databases are continuously updated, which means they can be used to track changes over time. Many countries make EPCs openly available, which means that methods based on EPCs are portable to other contexts.

However, EPCs provide an incomplete representation of the overall housing stock, since not all residential properties have an EPC. Un-corrected analyses using EPCs could produce biased estimates due to missing data and selection bias.

To our knowledge, no study has used EPCs to explore the geographic distribution of wood burning heat sources. The key objectives of this study were to:

- Use novel open data linkages to understand the geographic and socio-economic distribution of residential wood burning heat sources in two European countries (England and Wales)
- Develop and benchmark a transparent, portable tool to allow local policymakers to target interventions aimed at reducing emissions from residential wood burning

We used data from 26 million EPCs to map the distribution of wood burners in England and Wales. We evaluated the representativeness of the EPC dataset against a database of all residential properties in England and Wales and developed a method to correct for missing data in EPCs using Census data. Next, we estimated the prevalence and concentration of wood burners by Lower layer Super Output Area (LSOA). We evaluated our measure against PM_2.5_ measurements from the UK national air pollution monitoring network. To examine the association between LSOA-level prevalence of wood burners and socio-economic factors, we leveraged other open data on LSOA deprivation, ethnicity, and age. We described changes in the prevalence of wood burners since 2009. To supplement the main manuscript, we provide a publicly available report featuring interactive maps of the distribution of wood burners in England and Wales, available at: https://storymaps.arcgis.com/stories/9231a18627b94b3a80e4f33fb3b4a9fd.

## 2 Methods

A schematic overview of our methodology for the data cleaning, linkage, and final outputs is presented in Supplementary Figure 1.

### 2.1 Data

#### 2.1.1 The energy performance certificate dataset

Energy Performance Certificates (EPCs) are used in many countries to monitor the energy efficiency of the housing stock (Department for Levelling Up, Housing & Communities, 2024). EPCs are an information tool introduced under the 2002 EU Energy Performance of Buildings Directive, and contain information on a building’s energy efficiency alongside details on housing characteristics including property type, tenure, and primary and secondary heating source.

In England and Wales, a valid EPC certificate has been legally required at point of build, sale, or private rental since 2008. Accredited energy assessors generate the certificates, which are valid for ten years following inspection. The EPC database is updated monthly in England and Wales. The EPC dataset includes unique property reference numbers (UPRNs), which are unique identifiers for every addressable location in the UK.

#### 2.1.2 Other data sources

We used the 2019 Index of Multiple Deprivation (IMD) to measure area-level socio-economic status. The IMD is a measure of social deprivation determined at the LSOA level for England (Noble et al., 2019) and Wales (Welsh Government, 2022). The IMD is a combined measure based on weighted indicators of income, employment, education, health, crime, and the environment. Higher IMD scores indicate a higher level of social deprivation.

We extracted data from the 2021 Census for England and Wales to describe ethnicity at LSOA level, and data from the 2011 Census (the most recent available data) to assign urban/rural status to LSOAs (Office for National Statistics, 2022; Department for Environment, Food and Rural Affairs, 2021b). LSOAs were defined as rural if they were outside settlements with more than 10,000 residents. We used data from the Office for National Statistics’ population estimates to derive the mid-2022 estimates for median age by LSOA.

To evaluate the representativeness of the EPC dataset against the housing stock in England and Wales, we accessed data from the Ordnance Survey (OS) AddressBase. The OS AddressBase is a database linking around 29 million residential postal addresses to UPRNs, providing a complete list of active residential addresses in the UK. The OS AddressBase contains granular classification codes for property type, which can be mapped onto Census 2021 property type classifications.

We evaluated our high-resolution maps of residential wood burners against existing maps derived from the UK National Atmospheric Emissions Inventory (NAEI) and London Atmospheric Emissions Inventory (LAEI). The NAEI is a database of annual UK emissions estimates for air pollutants including PM_2.5_ (Tsagatakis et al., 2024). The NAEI generates 1 km × 1 km grid square estimates of PM_2.5_ emissions from residential wood burning, combining AddressBase data, 2011 Census data on housing and central heating type within small areas, a regional energy consumption model, and a 2020 household survey. The LAEI is a separate database of emissions estimates by grid square for Greater London and nearby areas within the M25 motorway which incorporates additional data sources available within London (Greater London Authority, 2023).

Finally, we downloaded hourly air quality data from three national air quality monitoring networks in the UK: the Automatic Urban and Rural Network (AURN), Air Quality England, and Air Quality Wales. The AURN is the main network used for compliance reporting against the UK’s Ambient Air Quality Directives (Department for Environment, Food and Rural Affairs, 2024a). Air Quality England and Air Quality Wales are locally-managed networks which are not part of the national monitoring network (Air Quality England, 2020; Air Quality in Wales, 2024). We accessed air quality data via the ‘openair’ R package (Carslaw and Ropkins, 2012).

### 2.2 Geographical hierarchies in the UK

We selected four geographical levels to report our main findings: LSOAs, Electoral Wards, LADs, and regions. We selected these geographies because they are the main reporting geographies for other data sources such as socio-economic characteristics, and because they are the main geographical units used for policymaking. For example, Local Authorities are responsible for local air quality controls and setting SCA boundaries.

LSOAs are UK Census geographies. In 2021 there were 33,755 LSOAs in England and 1,917 in Wales. LSOAs comprise between 400–1,200 households, and have a population between 1,000–3,000 people.

Electoral Wards, LADs, and regions are administrative geographies. In 2021, there were 6,980 Electoral Wards in England and 852 in Wales. Wards were nested within 318 LADs in England and 22 Unitary Authorities in Wales (LADs hereof). In England, LADs are nested within nine former government office regions, which are the highest tier of sub-national division.

### 2.3 Data cleaning and linkage

We downloaded all 27,491,063 EPCs from January 2009 to February 2025 and removed duplicated entries. We used UPRNs to link EPCs to higher level geographies (LSOA, Ward, LAD, and region) using the National Statistics UPRN Lookup for Great Britain as of May 2022 (Office for National Statistics, 2024). We linked retained records to LSOA-level data including IMD score, ethnicity, median age, and rural–urban status.

The final EPC dataset contained 26 million records, among which there were 18.7 million unique properties. Further details on the data cleaning procedure are available in Supplementary Appendix A.

### 2.4 Derived variables

We generated binary variables for whether a property’s primary or secondary heat source was a solid fuel or wood fuel heat source using keyword searches. A list of keyword searches used is available in Supplementary Appendix B.

Properties can have multiple EPCs. We generated a binary variable indicating whether an EPC was the most recent for that property. In our cross-sectional analyses and maps, we used the subset of most recent EPCs.

We re-categorised the property type and tenure variables in the EPC dataset to match the categories defined in the 2021 Census. Property type was defined as one of ‘detached’, ‘semi-detached’, ‘terrace’, ‘flat’, or ‘other accommodation’. Tenure was defined as one of ‘new build’, ‘owner-occupied’, ‘rented (private)’, or ‘rented (public)’.

We defined two outcome variables — the prevalence and the concentration of solid fuel and wood fuel heat sources by statistical geography (e.g. LSOA). The prevalence was defined as the percentage of most recent EPCs for houses in each geography which had a solid fuel or wood fuel heat source as the main or secondary heat source. We classified properties defined as ‘detached’, ‘semi-detached’, and ‘terrace’ in the 2021 Census as houses. We restricted the prevalence variable to houses since other accommodation types such as flats generally do not have capacity for wood burners, and had a very low prevalence of wood burners in previous surveys (Department for Environment, Food and Rural Affairs, 2020).

The concentration variable was defined as the number of most recent EPCs in each geography which had a solid fuel or wood fuel heat source as main or secondary heat source, divided by the total area of the geography in km^2^. The concentration is defined in relation to a geographic area as opposed to a specific population (houses). Therefore, we included all property types including flats. We used our concentration as a proxy variable for monitor-level wood burning emissions exposures in our comparisons with the NAEI methodology.

### 2.5 Spatiotemporal patterns in residential wood burning

Using EPCs, we estimated the percentage of properties with a wood fuel or solid fuel heat source by property type and LSOA. To mitigate uncertainty due to small sample size, we excluded LSOA–property type pairs where there were fewer than 20 EPCs for that LSOA–property type pair. This provided an estimate of the prevalence of wood and solid fuel heat sources in EPCs by LSOA and property type.

We used 2021 Census data to derive the total number of properties within LSOAs by property type. We estimated the total number of properties with wood fuel and solid fuel heat sources by property type by multiplying the estimated prevalence from EPCs with the number of properties of each property type from the Census. Finally, we aggregated the estimated number of properties with wood and solid fuel heat sources across property types to derive an estimate for the total number of properties with wood and solid fuel heat sources by LSOA.

To estimate the LSOA-level prevalence of wood fuel and solid fuel heat sources, we divided the estimated total number of properties with wood and solid fuel heat sources by the total number of properties in the LSOA. We derived the estimated concentration of wood and solid fuel heat sources by dividing the estimated total number of properties with a wood or solid fuel heat source by the area of the LSOA in km^2^. We compared our corrected estimates of the concentration and prevalence of wood burners to the uncorrected estimates using only EPCs. Mathematical details on our methodology are available in Supplementary Appendix C.

We produced choropleth maps of the estimated prevalence and concentration of wood burners by LSOA for England and Wales. To improve readability of the concentration maps, we truncated low and high extreme values to the 5th and 95th percentile values respectively.

To analyse temporal trends in the prevalence of wood burners in EPCs, we calculated the percentage of new EPCs issued each year which indicated presence of a wood burner for detached, semi-detached, and terraced houses. For this analysis, we included all EPCs, including properties with multiple EPCs. This is because the wood fuel status of a property can change over time, for example due to home renovations.

We visualised the change in prevalence of EPCs with wood burners in urban and rural areas stratified by IMD decile of the property’s LSOA. We also presented aggregate trends by property type and region.

Observed changes in prevalence could reflect changes to the housing stock (for example changes in building standards for new properties), changes in buying or renting behaviour due to preference shifts, or renovations to existing properties.

To explore whether renovations to existing properties are a relevant factor in driving trends in wood burner prevalence, we used the subset of properties with multiple EPCs. We reported the percentage of these properties which had a wood burner at time of first EPC, and for every subsequent EPC. We excluded results for properties with five or more EPCs due to small sample size.

### 2.6 Evaluating the wood burner concentration metric

To evaluate our wood burner concentration metric, we downloaded hourly PM_2.5_ measurements during 2022–2024 from the AURN, Air Quality England, and Air Quality Wales. We excluded 2020 and 2021 since these years may be unrepresentative of normal pollution patterns due to COVID-19. We also excluded years prior to 2020 as data from these years may not reflect the current distribution of wood burners.

We used data from 417 monitoring sites, of which 217 belonged to the Air Quality England network, 164 to the AURN, and 38 to the Air Quality Wales network. For our main analysis, we only used data from the AURN. This is because the AURN sites adhere to strict requirements based on national air quality directives, whereas locally-managed sites vary in data collection and quality practices (Department for Environment, Food and Rural Affairs, 2024a).

Drawing on previous work (Zalzal et al., 2024), we created a circular buffer with radius 1 km around each monitoring site and counted the estimated number of wood burners within the buffer radius. We considered two outcome variables — the average monitor-level PM_2.5_ and the difference between average PM_2.5_ at peak vs. non-peak burning times. We defined peak burning times to be between 7pm and 1am and non-peak burning times to be between 5am and 5pm. We restricted our analysis to monitoring sites classified as ‘Urban Background’ — urban monitoring sites where pollution is an integrated function of all sources upwind of the monitor.

We produced scatter plots of the correlation between the natural logarithm of the number of wood burners around a monitoring site and the two outcome variables. We plotted separate results for summer (June, July, and August) and winter (December, January, and February). We plotted the correlation between wood burner concentration and difference in peak vs. non-peak emissions separately for weekdays and weekends (defined as Saturday and Sunday).

For each scatter plot, we calculated the Spearman rank correlation coefficient (*R*) between the wood burner concentration variable and the outcome variable separately for the summer and winter seasons. We derived 95% confidence intervals for *R* using the non-parametric bias and skewness adjusted (BCA) bootstrap procedure with B = 10,000 replications (Efron, 1987). We also reported bootstrapped confidence intervals for the difference between the Spearman correlation coefficients in summer and winter. Our key assumption in generating confidence intervals using the bootstrap is that the sample of observed urban background monitoring sites is representative of the set of *possible* urban background monitoring sites.

To test the sensitivity of our results to the radius of the spatial buffer, we re-created the scatter plots using buffer radii of 500 m and 2 km. To further probe the robustness of our results, we plotted the correlation between concentration of wood burners and the outcome variables using urban background sites from all three monitoring networks. To test whether our concentration metric is simply a proxy for housing density, we produced the same plots replacing the *x*-axis variable with the total number of houses in the EPC data within a 1 km radius of the monitor.

We evaluated our concentration metric against 1 km^2^ grid square wood burning emissions estimates from the NAEI using AURN urban background sites. For each AURN urban background site, we counted the estimated number of wood burners in the site’s grid square using the EPC data. We produced the scatter plots for our grid square concentration metric, and compared them to the same scatter plots using the estimated grid square annual wood burning emissions from the current NAEI inventory. Whilst the EPC-based concentration metric does not attempt to measure actual PM_2.5_ emissions, the goal of this comparison was to test its utility as a tool to predict daily levels and peaks in PM_2.5_ emissions relative to the NAEI inventory.

### 2.7 Associations between prevalence of wood burners, housing characteristics, and socio-economic indicators

To examine the association between housing characteristics and wood burner prevalence, we produced tables summarising property characteristics separately for properties with and without wood burners. We also analysed the relationship between prevalence of wood burners and area-level socio-economic characteristics. To do so, we produced charts displaying the association between wood burner prevalence and LSOA-level IMD score and ethnic composition. Each chart was stratified by region, and averaged the explanatory variable by decile. We summarised LSOA-level socio-economic characteristics by decile of wood burner prevalence.

We built multivariate beta regression models to test the relative strength of the association between socio-economic indicators and prevalence of wood burners. Beta regression is a popular choice for modelling continuous outcomes bounded between zero and one, as with our prevalence metric. We used a logit link function, which means that the model coefficients can be interpreted as the association between a one unit change in the independent variable and the change in the logit of the expected value of the (beta-distributed) outcome variable.

In our first model we included all LSOAs. with independent variables LSOA median age, IMD score, urban/rural classification, SCA status, percentage of residents with a white ethnic background, and region indicators. We ran two further models stratified on the subset of urban and rural LSOAs respectively, including the same independent variables except for urban/rural status.

### 2.8 Reproducibility

All code used to produce the manuscript is available alongside replication instructions at: https://github.com/UCL-Wellcome-Trust-Air-Pollution/EPC_mapping_project_code. All non-EPC open data used to produce the manuscript is available at: https://zenodo.org/records/15453789.

All analyses were carried out in R version 4.4.1 on a Windows machine.

### 2.9 Role of the funding source

The funding source had no role in the study design, data collection and analysis, report writing, or the decision to submit this paper for publication.

## 3 Results

### 3.1 Representativeness of energy performance certificates

Out of the 28.6 million properties in the OS AddressBase, 18 million (63.0%) were matched to the EPC data. Flats were over-represented in EPCs, while detached, semi-detached, and terraced houses were under-represented. For example, 28.2% of properties in the EPC data were flats, compared to 23.8% in the OS AddressBase (Supplementary Table 1).

### 3.2 Spatiotemporal patterns in residential wood burning

Of the 18,708,520 unique EPCs, 1,586,345 (8.5%) had a solid fuel heat source. Of these, 1,368,802 (7.3%) were wood burners. The Census-corrected estimated concentration of wood burners was generally higher than that estimated using only EPCs (Supplementary Figure 2 Panel A). The estimated prevalence of wood burners closely matched the uncorrected estimates, suggesting that within property types EPCs are broadly representative of the OS AddressBase (Supplementary Figure 2 Panel B). Our Census-corrected prevalence estimates were 9.3% for solid fuels and 8.1% for wood fuel.

**Fig. 1:**
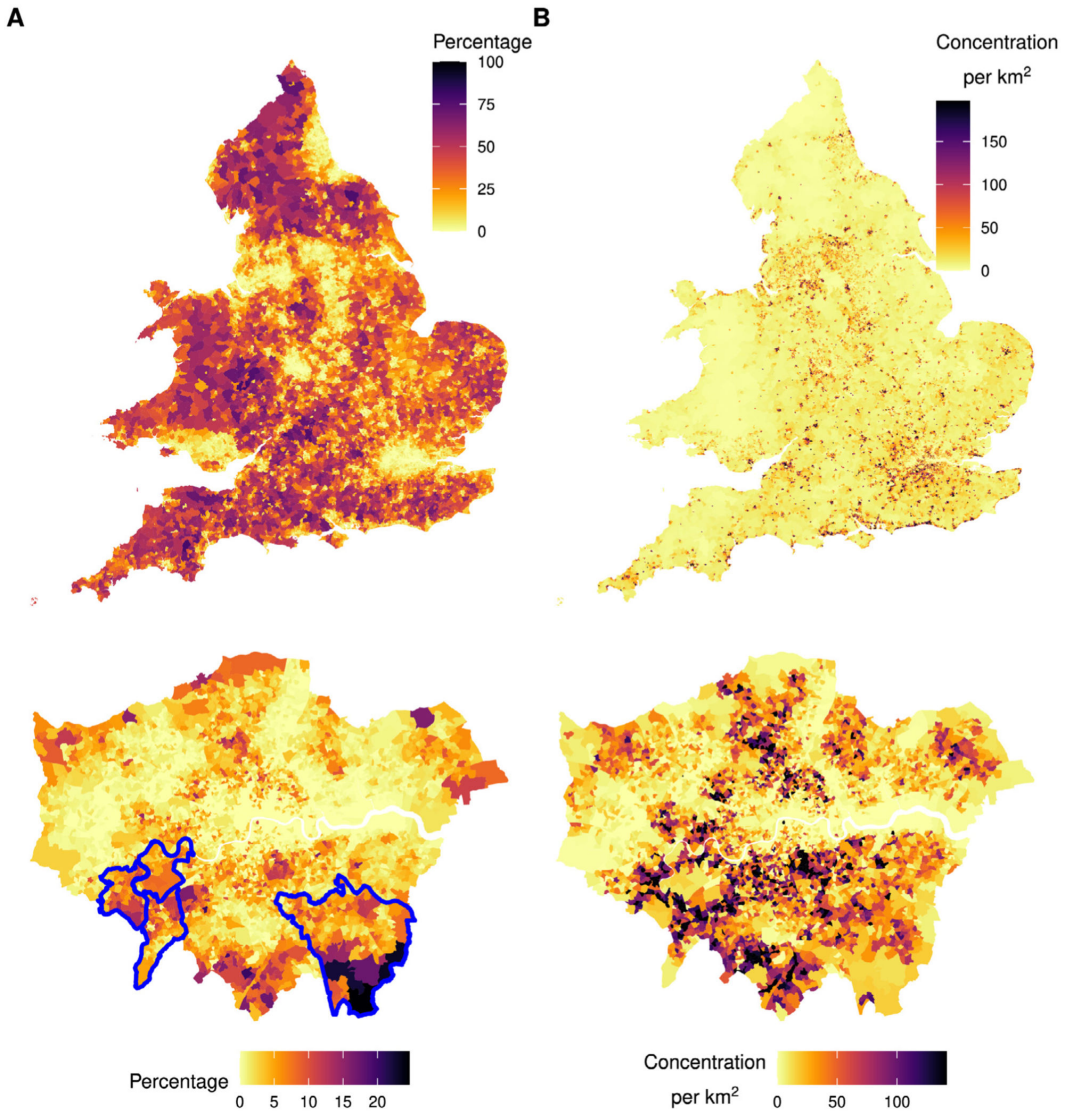
Estimated prevalence and concentration of wood fuel heat sources by LSOA, England and Wales. **A**. Top: Estimated prevalence of wood burners by LSOA, England and Wales. Bottom: Estimated prevalence of wood burners by LSOA, London. Local authorities of Richmond upon Thames, Kingston upon Thames, and Bromley highlighted in blue. **B**. Top: Estimated concentration of wood burners per km^2^ by LSOA, England and Wales. Bottom: Estimated concentration of wood burners per km^2^ by LSOA, London. Estimated concentration values were truncated at the 5th and 95th percentiles for mapping.

The prevalence of wood burners in EPCs was lowest in urban centres including Greater London, and Greater Manchester, and highest in rural areas in the South West, North West, and Wales (Fig. 1 Panel A, upper). There was a wide variation in the estimated prevalence of wood burners across LADs, ranging from 0.5% in the London borough of Newham to 43.7% in the Isles of Scilly.

Prevalence also varied substantially within urban areas. In Greater London (Fig. 1 Panel A, lower), the prevalence of wood burners was highest in the outer boroughs of Richmond upon Thames (6.1%), Kingston upon Thames (6.0%), Sutton (5.5%), and Bromley (5.5%), annotated in blue on the map.

The areas with the highest concentration of wood burners were in smaller urban centres and towns away from major cities (Fig. 1 Panel B, upper). The estimated concentration of wood burners was highest in the South Eastern LADs of Worthing (121.4 per km^2^), Norwich (105.7 per km^2^), and Reading (104.9 per km^2^).

There was substantial heterogeneity in the estimated concentration of wood burners within small urban areas (Fig. 1 Panel B, lower). The average concentration of wood burners across urban SCAs was 46.3 per km^2^ compared to 87.2 per km^2^ in non-SCA urban areas.

**Fig. 2:**
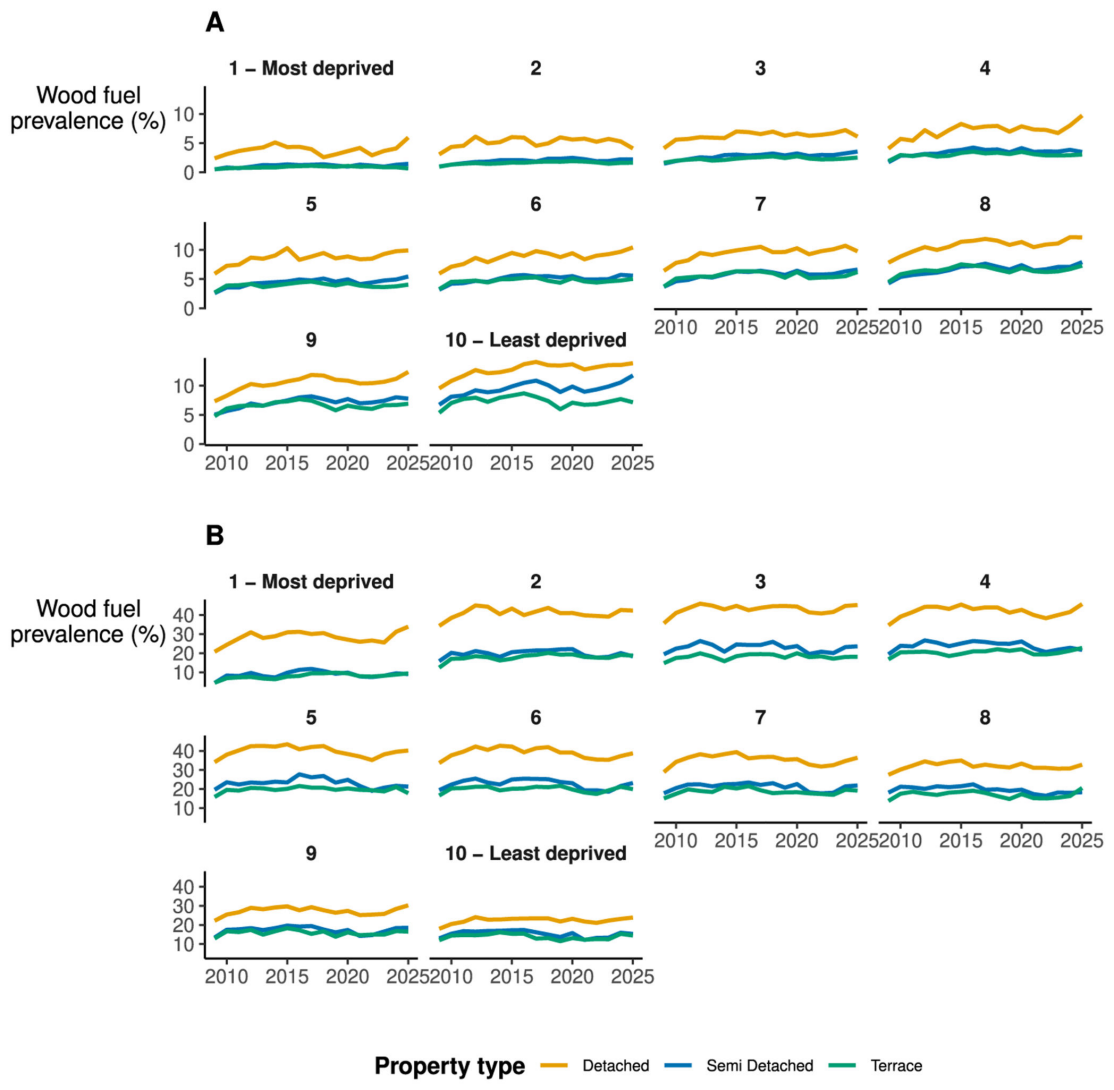
Prevalence of wood burners in EPCs by region and urban/rural status, January 2009–February 2025. **A**. Prevalence of wood burners in new EPC certificates from 2009–2025 for urban LSOAs, by property type and IMD decile. **B**. Prevalence of wood burners in new EPC certificates from 2009-2025 for rural LSOAs, by property type and IMD decile. The lowest IMD decile (1) corresponds to the most deprived areas, and the highest (10) corresponds to the least deprived. IMD deciles were calculated within urban/rural categories - for example, in **A**, deciles were calculated using only the subset of urban LSOAs. For each property type, IMD decile, and year, we calculated the percentage of new EPCs which indicated the presence of a wood burner. We restricted the analysis to the sub-sample of detached, semi detached, and terraced houses, excluding flats and other accommodation types. We used all EPCs available in the dataset, including multiple EPCs for the same property.

In our temporal analysis, we found a secular increasing trend in the prevalence of wood burners in EPCs. From 2009–2024, the prevalence of wood burners in new EPCs increased from 7.0% to 10.3%. The largest absolute increase was in detached homes, where prevalence rose from 15.2% in 2009 to 20.5% in 2024. In urban LSOAs, prevalence increased across all IMD deciles (Fig. 2).

Temporal trends in the prevalence of wood burners were broadly consistent across IMD deciles and property types, though less deprived LSOAs saw a larger absolute increase, due to higher baseline prevalence of wood burners.

In rural areas, wood burners were more prevalent in lower and middle deprivation deciles. As in urban LSOAs, prevalence was highest in detached houses. Compared to urban areas, trends in wood burner prevalence in rural areas remained roughly static over time.

We found evidence that renovations to existing properties are contributing to the increasing prevalence of wood burners (Supplementary Table 3). Among properties with multiple EPCs, prevalence of wood burners increased with each subsequent EPC the property received. For example, among the roughly 700,000 detached properties with two EPCs, 19.4% had a wood burner at the time of first EPC compared to 24.3% at time of second EPC.

### 3.3 Evaluating the wood burner concentration metric

**Fig. 3:**
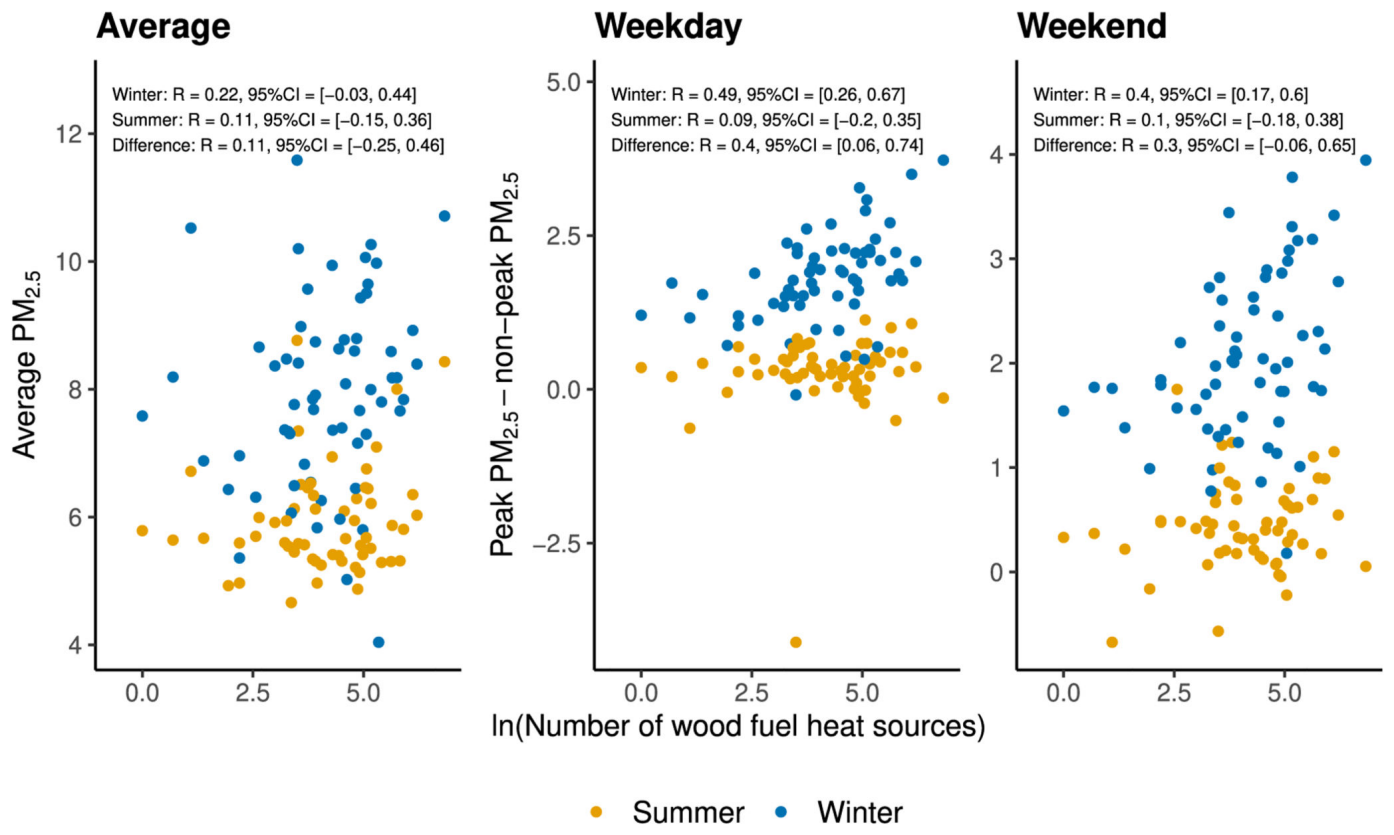
Scatter plot of log estimated wood burners against PM_2.5_ at AURN urban background monitoring sites. Left: Scatter plot of log estimated wood burner concentration against mean PM_2.5_ at monitoring sites. Centre: Scatter plot of log estimated wood burner concentration against difference in weekday mean PM_2.5_ measurements at peak vs. non-peak burning times. Right: Scatter plot of log estimated wood burner concentration against difference in weekend mean PM_2.5_ measurements at peak vs. non-peak burning times. Peak burning times were defined as 7pm–1am, and non-peak times as 5am–5pm. Weekends were defined as Saturday and Sunday. The concentration of wood burners was estimated as the number of properties with a wood burner in the EPC data within a 1 km circular radius of each monitoring site. We used sites classified as ‘Urban Background’ from the AURN. The scatter plots are stratified into summer and winter observations. Each chart reports the central estimate and 95% confidence interval for the Spearman correlation coefficient *R* separately for summer and winter. We calculated the 95% confidence intervals using the BCA bootstrap method with 10,000 replications. We also report bootstrap 95% confidence intervals for the difference between the summer and winter correlation coefficients.

The log concentration of wood burners was positively correlated with differences in peak vs. non-peak PM_2.5_ in winter, but not in summer (Fig. 3). On winter weekdays, the central estimate of the Spearman correlation was *R* = 0.49, with a BCA bootstrap 95% confidence interval of [0.26, 0.67]. On summer weekdays, the corresponding central estimate for *R* was 0.09 (95% CI [−0.2, 0.35]). The confidence interval for the difference between the winter and summer correlation coefficients was [0.06, 0.74]. Our central estimates for the correlation between log wood burner concentration and average PM_2.5_ were smaller, and both confidence intervals covered zero.

Expanding our data to urban background sites from all three monitoring networks, we found quantitatively similar results to our analysis using only the AURN (Supplementary Figure 6). Correlations between log wood burner concentration and differences in peak vs. non-peak PM_2.5_ were robust to buffer radii of 500 m and 2 km (Supplementary Figures 7–8). The correlation between log wood burner concentration and average PM_2.5_ varied by the choice of buffer radius — we observed a stronger correlation for wider buffer radii.

The EPC-based measure of housing density was weakly correlated with differences in peak vs. non-peak PM_2.5_ (Supplementary Figure 9). The point estimates for the correlation between log housing density and differences in PM_2.5_ between peak vs. non-peak burning times were *R* = 0.06 (95% CI [−0.19, 0.33]) on winter weekdays and *R* = 0.19 (95% CI [−0.06, 0.43]) on winter weekends.

Using grid square data from the NAEI, we continued to find evidence for a positive correlation between our measure of wood burner concentration and differences in measured PM_2.5_ at peak vs. non-peak burning times in winter (Supplementary Figure 10). The point estimates for the correlation between log wood burner concentration and differences in peak vs. non-peak average PM_2.5_ in winter were *R* = 0.47 (95% CI = [0.28, 0.63]) on weekdays and *R* = 0.35 (95% CI = [0.14, 0.53]) on weekends.

The implied grid square distribution of wood burning emissions from the existing NAEI inventory was poorly correlated with both average measured PM_2.5_ and differences in peak vs. non-peak average PM_2.5_ (Supplementary Figure 11). In winter, the point estimates for the correlation between NAEI-implied wood burning emissions and differences in peak vs. non-peak PM_2.5_ were 0.24 (95% CI = [0.03, 0.42]) on weekdays and 0.12 (95% CI [−0.09, 0.31]) on weekends.

### 3.4 Associations between prevalence of wood burners, housing characteristics, and socio-economic indicators

Properties with wood burners were more likely to be detached, owner-occupied properties (Table 1). Most wood burners were classified as secondary heat sources — 0.3% of properties had a wood burner as the primary heat source, whereas 7.1% had a wood burner as secondary heat source.

Low social deprivation was associated with higher prevalence of wood burners. The average IMD score in LSOAs in the lowest wood burner prevalence decile was 36.6, compared to 13.4 in the highest prevalence decile (Table 2). LSOAs with a higher prevalence of wood burners also had older populations. The median age in LSOAs in the lowest wood burner prevalence decile was 33.7 years, rising across every subsequent decile to 51.2 years in the highest prevalence decile. Prevalence of wood burners was also positively correlated with the proportion of LSOA residents identifying as having a white ethnic background.

**Fig. 4:**
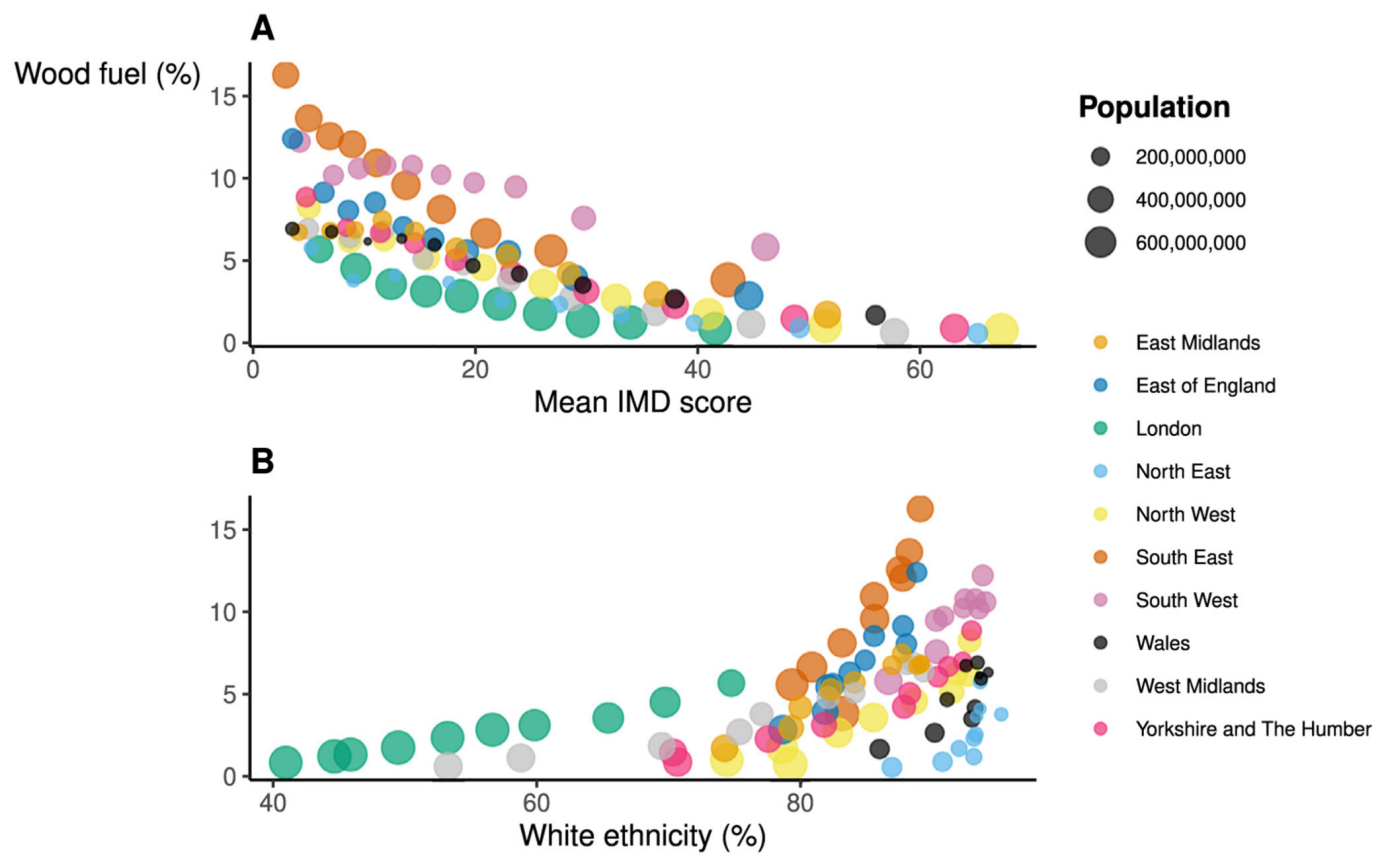
Association between social deprivation, ethnicity, and prevalence of wood fuel heat sources in urban LSOAs, England and Wales. **A**. Association between mean IMD score and prevalence of wood burners – urban LSOAs. Within regions, we grouped urban LSOAs by IMD decile and calculated the mean IMD score and prevalence of wood burners for each region-IMD decile pair. Each point on the plot represents the mean IMD score and WF prevalence for a given region and IMD decile. The size of the point corresponds to the total population within that region and IMD decile. **B**. Association between percentage of people from a white ethnic background and prevalence of wood burners – urban LSOAs. We followed the same method as in **A** to generate the plot.

Across all regions, wood burner prevalence in urban LSOAs was negatively associated with social deprivation and positively associated with the percentage of residents identifying as ethnically white (Fig. 4). The relationship between socio-economic characteristics and wood burner prevalence was different in urban and rural LSOAs (Supplementary Figures 13–15). In rural areas, there was no clear univariate relationship between social deprivation and prevalence of wood burners (Supplementary Figure 13).

Results from multivariate beta regression supported the univariate associations between socio-economic characteristics and wood burner prevalence. Urbanicity, SCA restrictions, and IMD score were all negatively associated with prevalence of wood burners, while median age and percentage of residents identifying as white were positively associated (Supplementary Table 4). For example, the central estimate for the IMD score coefficient was −0.012 (95% CI [-0.013, −0.011]), which implies that a one unit increase in social deprivation score (which indicates increasing social deprivation) was associated with a −0.012 unit change in the logit of the expected value of the LSOA-level wood burner prevalence. All coefficients were large relative to their standard error, with confidence intervals excluding zero.

Restricting our model to urban LSOAs, we identified the same directionality of association between wood burner prevalence and SCA restrictions, median age, percentage of residents identifying as ethnically white, and IMD score. However, the strength of the relationship was weaker for median age, and stronger for the ethnicity and social deprivation variables (Supplementary Table 5). In rural LSOAs, the prevalence gradient was stronger with respect to age and weaker with respect to social deprivation and ethnicity (Supplementary Table 6).

## 4 Discussion

### 4.1 Summary

This study was motivated by the growing body of literature linking residential wood burning to air pollution and adverse health impacts, alongside a relative lack of evidence on the geographic and socio-economic distribution of wood burning in high-income countries. Existing wood burning inventories are often unsuitable for targeting public health interventions, highlighting the need for more detailed spatial data.

Using EPCs, we found substantial variability in the prevalence and concentration of wood burners across England and Wales. While wood burners were more prevalent in rural areas, they were more densely concentrated in populated urban settings. A notable finding was the high concentrations within historic SCAs. Even within small urban areas, the distribution of wood burners varied considerably. At the LSOA level, the prevalence of wood burners was positively associated with older median age of residents, low social deprivation, and a higher percentage of residents identifying as ethnically white.

‘Real world’ use field studies have shown that wood stove usage leads to substantial increases in indoor air pollution. For example, a UK field study of households with SCA-exempt appliances found that daily average household indoor PM_2.5_ levels during stove use were nearly three times higher than during non-use (Chakraborty et al., 2020). Wood burning has also been linked to increases in urban ambient outdoor air pollution during popular burning times including winter evenings and weekends (Font et al., 2022).

Systematic reviews in higher-income countries identify links between wood smoke exposure and adverse health outcomes, particularly for children (Guercio et al., 2022, 2021). A large prospective US cohort study of 50,226 women reported that using wood for heating for more than 30 days per year increased the incidence of lung cancer by 68% (95% CI: 27%–220%) (Mehta et al., 2023). A recent time-series study in London found that short-term exposure to increased levels of wood smoke was associated with an increase in risk of respiratory mortality of 1.70% per interquartile range increase in three-day atmospheric concentration of wood burning carbon (95% CI: 0.64–2.27%) (Piper et al., 2024).

Against this backdrop, we identified an important dichotomy between the likely sources of wood smoke exposures in urban and rural areas. In urban areas, wood burners are highly concentrated but uncommon at the household level, implying that ambient outdoor air pollution is the primary exposure source. By contrast, indoor emissions are the primary exposure source in rural areas where wood burners are prevalent, but sparsely distributed.

Our findings with respect to the socio-economic characteristics of residential wood burning are consistent with evidence on from two recent surveys of residential solid fuel burning in the United Kingdom (first survey conducted 2018–19 (N = 46,729), updated 2022–23 (N = 39,359)) (Department for Environment, Food and Rural Affairs, 2020, 2024b). The 2022–23 survey estimated that 11.7% of UK households burned solid fuels indoors. In the highest ‘AB’ social grade (a socio-economic classification based on employment type) the prevalence was 15.7%, compared to 7.5% in the lowest ‘DE’ social grade.

Comparing estimates of the overall prevalence of solid fuel burning from the 2018–19 and 2022–23 surveys also indicates an increase in the prevalence of indoor solid fuel burning in recent years. In the 2018–19 survey, 8.0% of respondents said that they burned solid fuels indoors, compared to 11.7% in 2022–23. These results are consistent with our analysis of the prevalence of wood and solid fuel heat sources in EPCs, suggesting that our method is able to capture underlying temporal trends.

We found clear differences in the socio-economic characteristics of wood burning between urban and rural areas, particularly with respect to social deprivation. Previous research has also highlighted socio-economic differences between urban and rural wood burning. For example, a study in Quebec found that median household income was positively associated with ownership of fireplaces (predominantly found in urban homes) and negatively associated with ownership of wood stoves (predominantly found in rural homes) (Zalzal et al., 2024). More generally, the reasons for burning wood vary between urban and rural areas, with wood more commonly used as a primary heating source in rural areas (Department for Environment, Food and Rural Affairs, 2020).

Our finding that wood burning heat sources are most prevalent in areas with low social deprivation is interesting in the context of a recent trend analysis of lung cancer in non-smokers (LCINS) in the UK (Rait and Horsfall, 2020). The study found that, contrary to overall trends, LCINS incidence increased for women living in the least deprived areas between 1998–2018. Women in the UK spend more time at home (on average) than men. Therefore, one explanation for rising rates of LCINS for women in affluent areas could be increased exposure to harmful pollutants from residential combustion.

Assuming that our EPC-based metric is a good proxy for wood burning PM_2.5_ emissions, we highlight the potential for environmental injustice. In affluent urban areas, households almost always have alternative heat sources available. In a segmentation analysis of the 2018–19 household solid fuel burning survey, fewer than 10% of households were classified as ‘necessity’ burners, where solid fuels were the main source of heating (Department for Environment, Food and Rural Affairs, 2020). The proportion of ‘necessity’ burners was lowest in the highest social grades and in urban households.

Spatial variability in emissions sources can generate socio-economic inequities in air pollution exposures (Batisse et al., 2025; Venter et al., 2023). Discrepancies in air pollution exposures can have lasting effects — historic air pollution patterns have been linked to the East–West deprivation gradient in formerly industrial cities in England and Wales (Heblich et al., 2021). In urban areas where wood burners are highly concentrated, local populations are potentially being exposed to high levels of air pollution from wood burning which is largely discretionary.

### 4.2 Comparison against existing methods for high-resolution mapping of residential wood burning

The NAEI wood burning inventory also identifies wood burning hotspots in urban areas (Tsagatakis et al., 2024). However, the NAEI hotspots are different to the areas with the highest concentration of wood burners identified using EPCs. NAEI maps identify northern English towns with relatively higher levels of social deprivation as emitting the highest quantity of PM_2.5_ from residential wood burning. By contrast, we identified affluent towns in southern England as having the highest concentration of wood burners. However,direct evaluation of our findings against the NAEI is challenging, since the methods target different quantities. The NAEI maps attempt to quantify the spatial distribution of wood burning emissions, whereas our method quantifies the spatial distribution of wood burning heat sources.

High-resolution mapping of residential wood burning is challenging, since wood burning is a diffuse emissions sector. Existing residential wood burning maps are typically derived from national wood burning emissions inventories. In the absence of point source activity data, these inventories typically integrate aggregated activity data (e.g. national emissions estimates) with various spatial proxy data sources including population density, building registries, census data, and household surveys on wood consumption (Karvosenoja, 2008; Plejdrup et al., 2016; Lopez-Aparicio et al., 2018; Kuenen et al., 2022; Borchers-Arriagada et al., 2024; US Environmental Protection Agency, 2023; Zalzal et al., 2024; Tsagatakis et al., 2024).

Buildings registries and census data serve a similar function to our EPC data in that they are used to proxy the total number of wood burning heat sources within a specified geography. Household surveys are often used to proxy the spatial intensity of wood burning activity — for example via estimates of the quantity of wood fuel consumed by responding households (Tian et al., 2004; Gonçalves et al., 2012; Grythe et al., 2019; US Environmental Protection Agency, 2023). Less frequently, surveys have also been used to calibrate disaggregated estimates of wood fuel users against higher-level geographies (Tsagatakis et al., 2024).

Our EPC-based method has several advantages relative to existing inventory-based methods as a tool to identify potential wood burning hotspots. Existing emissions maps combine multiple data sources with varying spatial granularity. For example, although census data is typically available at high geographic resolution, survey estimates are typically available at lower resolution due to sample size constraints.

To deal with spatial mismatch in input data, current methods typically present gridded estimates of wood burning emissions based on various smoothing assumptions. Smoothed grid square estimates may be insufficient to capture the high spatial variability in wood burning heat sources, and can be sensitive to the granularity of the input data (Vedrenne et al., 2016). Furthermore, grid squares do not directly correspond to administrative or electoral boundaries which are used in local policymaking.

A major benefit of our EPC method is its high spatial granularity. This advantage is particularly pronounced in urban areas — the average area of a London LSOA is 0.32 km^2^, compared to the 1 km^2^ grid squares available via the NAEI. This increased spatial granularity is possible precisely because we do not rely on low-resolution input data sources, and therefore make minimal spatial smoothing assumptions. EPCs can also be easily linked to administrative boundaries relevant for policymaking.

A further advantage of EPCs is that the dataset is updated continuously as new EPCs are generated. In the UK, the EPC dataset is updated monthly. Many existing methods rely on data sources which are infrequently updated (e.g. Census data) or ‘point-in-time’ snapshots (e.g. one-off household surveys), which means that estimates may be out of date. By contrast, EPCs provide up-to-date information which can be used to track trends or outliers over time.

It is important to highlight that unlike national emissions inventories, our EPC-based method does not attempt to quantify the geographic distribution of PM_2.5_ emissions from residential wood burning. Our method relies on the geographic distribution of wood burning heat sources as a proxy variable for emissions. Our key assumption in this analysis is that, at least within small areas, our measure is indeed a good proxy for wood burning emissions.

In our validation exercise, the wood burning concentration metric was correlated with peaks in winter PM_2.5_ in urban areas, which gives some reassurance that our measure captures meaningful spatial variation in wood burning emissions. Interestingly, our concentration metric was better-correlated with peaks in winter PM_2.5_ than the existing NAEI maps. High-resolution emissions maps based on granular input data have also outperformed national emissons inventories in other contexts (Zalzal et al., 2024). However, our results should not be interpreted as estimates for the true geographic distribution of wood burning emissions in England and Wales.

### 4.3 Limitations

Our analysis has several important limitations. Using EPCs, we cannot reliably differentiate between different appliances or fuel types. Emissions factors vary substantially between appliances. For example, open fires emit around nine times as much PM_2.5_ per gigajoule burned as modern ‘Ecodesign’ closed stoves, which comply with EU energy efficiency standards (Department for Environment, Food and Rural Affairs, 2021a; European Union, 2024). Our concentration maps may over-represent potential emissions in SCAs, since these areas are subject to legal restrictions on the types of appliance and fuels that can be used. In the 2022–23 household burning survey, respondents living in SCAs were more likely to own solid or dual fuel range cookers, automatic pellet stoves, or biomass boilers compared to respondents outside of SCAs (Department for Environment, Food and Rural Affairs, 2024b).

EPCs are also not informative about usage intensity. Our solid fuel prevalence estimates were generally higher than the percentage of respondents who reported burning solid fuels in the household solid fuel burning survey. For example, we estimated that 12.8% of properties in the South East had a solid fuel heat source, whereas 9.3% of survey respondents in the South East reported using a solid fuel heat source for indoor heating. The discrepancy between our results and the survey estimates could reflect the fact that some households with solid fuel heat sources may choose not to use them. Previous work has found a higher intensity of wood burning in rural areas (Zalzal et al., 2024; Paunu et al., 2021). Our results could understate the relative importance of rural wood burning for ambient air pollution, since they do not model geographic differences in burning intensity.

EPCs remain valid for ten years, meaning that existing EPC data may be out of date. We found evidence of increasing prevalence of wood burners in properties with multiple EPCs. Reliance on out-of-date EPCs could lead us to systematically underestimate the prevalence of wood burners, particularly in areas where EPCs are updated infrequently.

EPCs are a biased sample of the UK housing stock. Our method to correct for missing EPC data relies on an assumption that within LSOAs and housing types, EPC missingness is uncorrelated with wood burner presence. Whilst we argue that this is a relatively weak assumption given the homogeneity of LSOAs, it is possible that there is still some residual selection bias.

Our validation exercise does not consider weather variables such as prevailing wind direction or wind speed, which could potentially confound the relationship between our proxy concentration measure and monitor-level PM_2.5_. For example, if the number of wood burners within buffer zones is correlated with their spatial distribution (e.g. in buffers with more wood burners, a higher proportion of those wood burners are located upwind of the monitor) then our method could give biased results by assuming a uniform distribution of heat sources around the monitoring site. However, these biases could only appear if the spatial distribution of wood burners within the buffer was systematically correlated with the weather variables. We argue that this is unlikely, given that we use a relatively large sample of monitoring sites and restrict our analysis to urban background sites.

Observed correlations with differences in peak vs. non-peak PM_2.5_ could reflect the influence of confounding pollutants — for example, air pollution from residential cooking. However, the weak relationship between housing density and differences in peak and non-peak PM_2.5_ provides some evidence that our measure extracts signal independent to the effects of these potential confounders.

### 4.4 Policy implications and future research

The high spatial heterogeneity in our estimated concentration of wood burners validates a targeted approach to intervention, particularly in urban areas where wood burners are highly concentrated. Policymakers could use EPCs to extend existing SCAs or create new ones. To-date, air quality policy efforts have largely focused on major cities and metropolitan areas. Our results highlight an important role for policy in smaller urban areas, where wood burners can be more highly concentrated than in cities. In rural areas, public health and primary care messaging should focus on the potential health risks from indoor wood smoke exposures, especially given that rural populations tend to be older.

Local topography and climate will influence how wood burning emissions are distributed, and hotspots for air pollution exposures may differ from hotspots for source emissions. EPC data on the geographic distribution of wood burning and solid fuel heat sources could be a valuable addition into routine air quality modelling. Current air quality monitoring networks are biased towards traffic emissions from major roads. EPCs could also be used to guide strategic expansion of monitoring networks into diverse residential areas to understand ambient air quality where people live.

To-date, there has been relatively little work to quantify the relationship between residential wood burning and socio-economic characteristics in high income countries. It is difficult to compare findings across contexts, since wood burning behaviours can vary substantially across countries (Paunu et al., 2021; López-Aparicio et al., 2017). Future work should seek to characterise the relationship between socio-economic characteristics and residential wood burning in other high income contexts.

Linkage of EPCs with air quality data and national health datasets could advance our understanding of wood smoke’s impact on health. Robust evidence is needed on the heath impacts of indoor air pollution exposures, which is particularly pertinent given the high prevalence of wood burners in rural areas (Department of Health and Social Care, 2022).

Our work highlights the potentially important confounding effects of age, social deprivation, urbanicity, and ethnicity on the relationship between wood smoke exposures and health. These factors are not routinely accounted for in existing epidemiological analyses (Rokoff et al., 2017; Guercio et al., 2022). Future studies seeking to understand the health impacts of wood smoke exposures should develop careful causal inference designs accounting for socio-economic confounding.

## 5 Conclusion

Residential wood burning represents a significant and growing public health concern, contributing to air pollution and potentially to a range of health risks. Monitoring and regulating this source is particularly difficult, as wood burning takes place within private households and is poorly captured by existing air quality monitoring networks. In this study, we used data from 26 million EPCs to characterise the geographic distribution of wood burners across England and Wales at fine scale. The size and granularity of the EPC data allowed us to detect substantial spatial heterogeneity in the concentration of wood burners, even within small urban areas. Linking EPCs to other open data sources, we described previously-understudied relationships between the prevalence of wood burners and socio-economic characteristics including age, deprivation, and ethnicity.

Our method relied on open data sources, and we generated a publicly-available, reproducible code base which can be used to fully replicate the analysis. Many countries make similar EPC data openly available, which means our method is portable to other contexts.

Public health officials could use EPCs to identify potential wood burning hotspots, target local awareness campaigns, or to extend legal restrictions on solid fuel burning. Our EPC-based concentration measure was better-correlated with winter peaks in urban PM_2.5_ than existing UK National Atmospheric Emissions Inventory maps, suggesting that EPCs could be a valuable addition to routine air quality modelling.

Our work highlights important issues of environmental justice. Wood burners were most concentrated in affluent urban areas, where households almost always have alternative heat sources available. Moreover, our results show the importance of accounting for socio-economic confounding effects in analyses of wood smoke exposures and health.

## Supplementary Material

**Appendix A. Supplementary data**

Supplementary material related to this article can be found online at https://doi.org/10.1016/j.envint.2025.109537.

Supplementary File

## Figures and Tables

**Table 1:** Property characteristics by presence of wood burner in England and Wales.

	Wood burner	
	No (N = 17,339,718)	Yes (N = 1,368,802)
Property type		
Detached	2,890,474 (17%)	679,637 (50%)
Flat	5,308,610 (31%)	26,946 (2.0%)
House Form Missing	53,891 (0.3%)	2,781 (0.2%)
Other accommodation	9,772 (<0.1%)	215 (<0.1%)
Semi Detached	4,295,286 (25%)	377,068 (28%)
Terrace	4,781,685 (28%)	282,155 (21%)
Tenure		
New build	738,240 (4.6%)	18,278 (1.4%)
Owner-occupied	8,707,524 (55%)	1,105,820 (85%)
Rented (private)	3,299,967 (21%)	165,375 (13%)
Rented (social)	3,143,415 (20%)	18,706 (1.4%)
Missing	1,450,572	60,623
Smoke Control Area		
No	8,427,370 (49%)	1,159,221 (85%)
Yes	8,912,348 (51%)	209,581 (15%)
Urban LSOA		
No	2,455,205 (15%)	746,353 (56%)
Yes	14,093,037 (85%)	594,822 (44%)
Missing	791,476	27,627

Note: We used the subset of unique properties in the EPC dataset. We excluded properties which had missing information on wood fuel presence. Each column shows the count of properties in the EPC database with the specified characteristic and wood fuel status, and the percentage of all properties of that wood fuel status with the specified characteristic. Missing values within characteristics are reported.

**Table 2:** Summary of socio-economic indicators by LSOA wood fuel prevalence decile.

Decile	Wood fuel prevalence (%)	IMD score	White ethnic background (%)	Median age	Urban (%)
1	0.1	36.6	56.3	33.7	99.8
2	0.7	34.6	70.6	37.0	99.1
3	1.5	28.6	79.0	39.1	97.5
4	2.5	24.4	82.0	40.4	96.1
5	3.8	21.0	84.2	41.5	94.0
6	5.5	17.9	86.7	42.8	91.9
7	7.9	15.9	89.1	44.2	86.3
8	11.5	13.9	90.9	45.5	79.6
9	18.5	12.2	93.0	47.5	62.8
10	40.1	13.4	96.2	51.2	17.5

Note: The table presents summary statistics on LSOA-level socio-economic indicators grouped by deciles calculated using the estimated prevalence of wood burners by LSOA. The first decile represents LSOAs with the lowest prevalence of wood burners, and the tenth decile represents LSOAs with the highest prevalence of wood burners.

## Data Availability

This study used data on all EPCs made available to registered researchers up to February 2025 under the Open Government Licence v3.0, including Ordnance Survey UPRNs. EPC data is available at: https://epc.opendatacommunities.org/. We used OS AddressBase data under an Ordnance Survey research licence. To facilitate replication of the analysis, we directly provide the summarised OS AddressBase data used to produce the final tables in the manuscript as part of our data files. All other data are from open or public data sources available under the Open Government Licence. All non-EPC open data used in the research are available here: https://zenodo.org/records/15453789. All R code used for the data cleaning, analysis, and reporting is available here: https://github.com/UCL-Wellcome-Trust-Air-Pollution/EPC_mapping_project_code. All analyses and data visualisations were created using R version 4.4.1.
