# Supplementary material for: High-resolution mapping of residential wood burning heat sources using Energy Performance Certificates: A case study of England and Wales
Source: Environ Int. Author manuscript; Available in PMC 2026 Jan 6. (PMC7618568; doi:10.1016/j.envint.2025.109537)
Supplement: Supplementary File [file EMS211737-supplement-Supplementary_File.pdf]

Supplementary information for "High-resolution mapping of domestic wood burning heat sources using Energy Performance Certificates: A case study of England and Wales"

Laura Horsfall*[1], Calum Kennedy[1], Nicola Shelton[2], Jonathon Taylor[3], John R Hurst[4], Matthew Loxham[5], Adam Dennett[6], and Pia Hardelid[7]

[1]Institute of Health Informatics, University College London, London, United Kingdom, NW1 2DA
[2]Department of Epidemiology and Public Health, University College London, London, United Kingdom, WC1E 7HB
[3]Faculty of Built Environment, Tampere University, Korkeakoulunkatu 7, Tampere, Finland, 33720
[4]UCL Respiratory, University College London, London, United Kingdom, NW3 2PF
[5]Institute for Life Sciences, University of Southampton, Southampton, United Kingdom, SO17 1BJ
[6]Bartlett Centre for Advanced Spatial Analytics, University College London, London, United Kingdom, W1T 4TJ
[7]Great Ormond Institute of Child Health, University College London, London, United Kingdom, WC1N 1EH

April 2025

*Corresponding author: laura.horsfall@ucl.ac.uk, +44 (0) 20 3549 5969

Table of contents

Summary **3**

Figures **4**

Tables **20**

A Data cleaning and linkage **26**

B Search terms for primary and secondary heat source **27**

C Technical appendix **28**

List of Figures

2 Corrected vs. uncorrected estimated concentration and prevalence of wood burners by LSOA . . . 6
3 Maps of estimated prevalence and concentration of solid fuel heat sources by LSOA, England and Wales . . . 7
4 Temporal trends in prevalence of wood burners in EPCs, by property type and region . . . 8
5 Map of EPC vs. LAEI implied distribution of wood burning emissions, London 9
6 Scatter plot of log estimated concentration of wood burners against $PM_{2.5}$ at AURN, Air Quality England, and Air Quality Wales monitoring sites . . . 10
7 Scatter plot of log estimated concentration of wood burners against $PM_{2.5}$ at AURN monitoring sites, using spatial buffer of 500m . . . 11
8 Scatter plot of log estimated concentration of wood burners against $PM_{2.5}$ at AURN monitoring sites, using spatial buffer of 2km . . . 12
9 Scatter plot of log estimated housing density against $PM_{2.5}$ at AURN monitoring sites . . . 13
10 Scatter plot of log estimated concentration of wood burners against $PM_{2.5}$ at AURN monitoring sites . . . 14
11 Scatter plot of NAEI-implied wood burning emissions against $PM_{2.5}$ at AURN monitoring sites . . . 15
12 Association between social deprivation, ethnicity, and prevalence of wood burners by region . . . 16
13 Association between social deprivation and prevalence of wood burners, by LSOA and urban/rural classification . . . 17
14 Association between ethnicity and prevalence of wood burners, by LSOA and urban/rural classification . . . 18

15 Association between age and prevalence of wood burners, by LSOA and urban/rural classification 19

List of Tables

1 Percentage of properties in the OS AddressBase and EPC dataset, by housing type 20
2 Property characteristics by presence of solid fuel heat source in England and Wales 21
3 Percentage of EPCs with a wood burner by property type and EPC number . . 22
4 Beta regression of prevalence of wood burners on LSOA-level characteristics . . 23
5 Beta regression of prevalence of wood burners on LSOA-level characteristics - urban LSOAs 24
6 Beta regression of prevalence of wood burners on LSOA-level characteristics - rural LSOAs 25

Summary

This file contains supplementary information for the manuscript "High-resolution mapping of domestic wood burning appliance hotspots using Energy Performance Certificates: A case study of England and Wales". Figure 1 presents a schematic overview of the methodology used to clean and link the various data sources, and the final outputs produced. Figure 2 compares our corrected LSOA-level estimates for concentration and prevalence of wood fuel heat sources to the concentration and prevalence estimated using only EPC data. Figure 3 displays maps of the concentration and prevalence of solid fuel heat sources in England and Wales, and in London. Figure 4 shows temporal trends in the prevalence of wood fuel heat sources in new EPCs from 2009-2024 by housing type and region.

Figure 5 compares the estimated concentration of wood burners in London using EPCs to LAEI emissions maps from domestic wood burning. Figure 6 presents the relationship between log concentration of wood burners (estimated using EPCs) and two outcome variables - mean $PM_{2.5}$ measurements (left panel) and the difference between peak and non-peak $PM_{2.5}$ emissions (separated into weekdays (centre) and weekends (right)). Each plot is stratified into summer and winter measurements, and uses data on monitoring sites classified as 'Urban Background' from the AURN, Air Quality England, and Air Quality Wales.

Figure 7 presents the same plots as in Figure 6, using only AURN urban background monitoring sites and using a spatial buffer of 500m around each monitor. Figure 8 extends the spatial buffer to a 2km radius around the monitoring sites. Figure 9 presents the same plots replacing the x-axis with the log housing density around each monitor and restricting to AURN sites. Figure 10 presents the same plots using the subset of 'Urban Background' AURN sites in the NAEI. Here, the concentration of wood burners is defined as the number of wood burners in the corresponding 1km x 1km grid square of the monitoring station. Figure 11 replaces the

x-axis variable with the implied grid square annual wood burning emissions using the existing NAEI method.

Figure 12 displays the associations between mean IMD score and prevalence of wood fuel heat sources, and between percentage white ethnicity and prevalence of wood fuel heat sources by LSOA. Figure 13 - Figure 15 display scatter plots of the association between wood fuel prevalence and various socio-economic factors by LSOA. Colour is used to distinguish between urban and rural LSOAs.

Table 1 assesses the representativeness of the EPC data against the OS AddressBase. Table 2 provides summary statistics of the EPC dataset stratified by access to a solid fuel heat source. Table 3 displays the prevalence of wood burners at each EPC for properties with multiple EPCs. Table 4 presents results from a beta regression of LSOA-level prevalence of wood burners against socio-deomgraphic characteristics. Table 5 and 6 present results from the same beta regression restricted to the subset of urban and rural LSOAs respectively.

Appendix A describes the data cleaning and linkage in more detail. Appendix B gives further details on the search terms used to identify wood and solid fuel heat sources in the EPC data. Mathematical details of the method used to correct for missing data and selection bias in EPCs using Census data is presented in Appendix C.

Figures

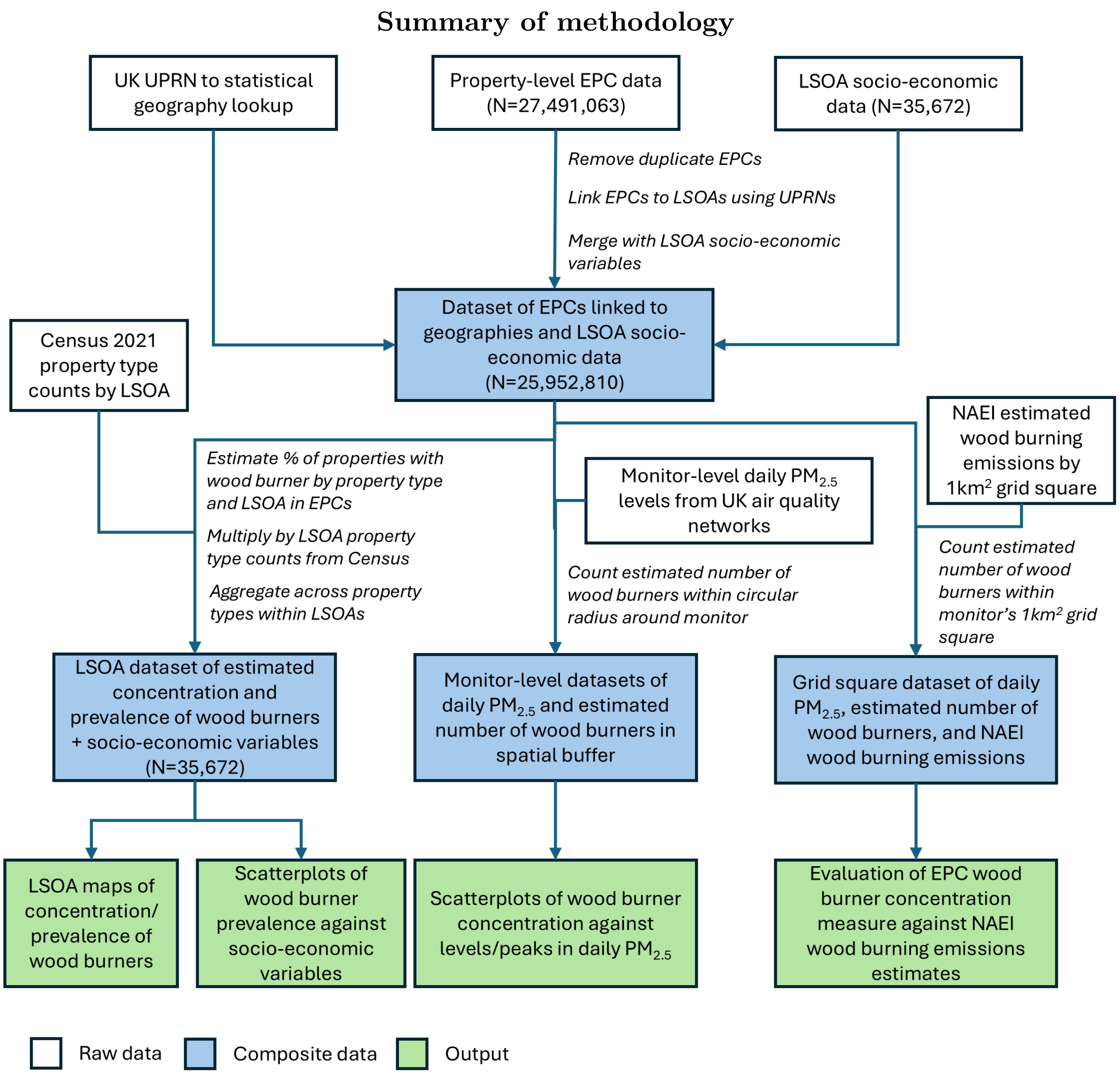

Figure 1: Flowchart of methodology for data cleaning, linkage, and final output

Corrected vs. uncorrected estimated concentration and prevalence of wood burners by LSOA

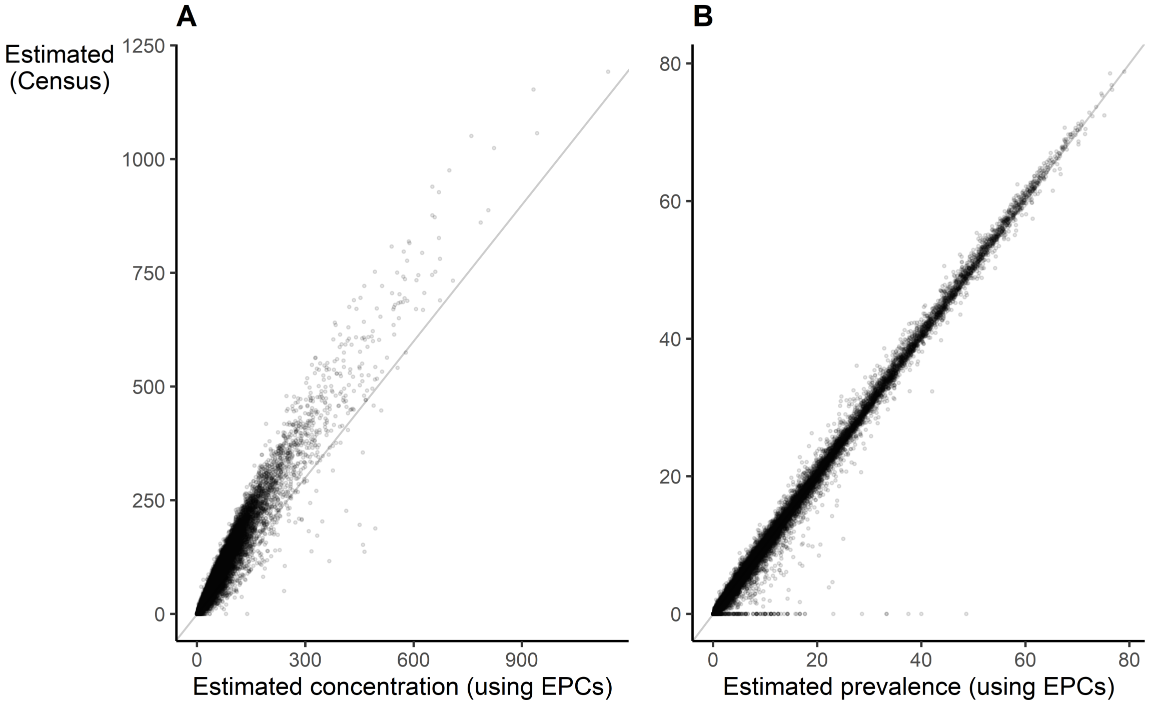

Figure 2: **A -** Scatter plot of estimated concentration of wood fuel heat sources per km^2 against estimated concentration using Census-based correction method by LSOA. **B -** Scatter plot of estimated prevalence of wood fuel heat sources against Census-based estimated prevalence by LSOA. Solid lines are 45 degree lines.

Maps of estimated prevalence and concentration of solid fuel heat sources by LSOA, England and Wales

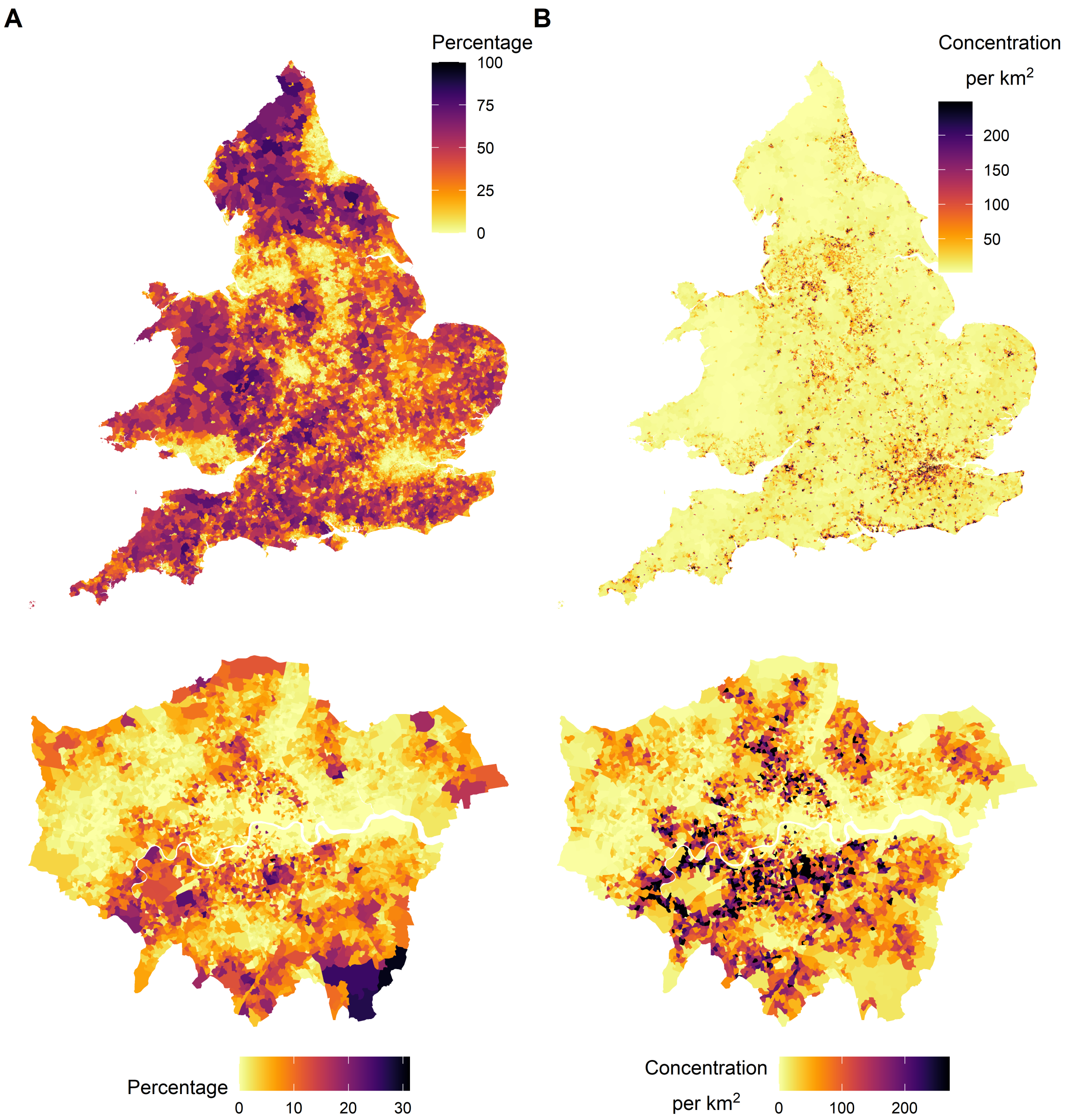

Figure 3: **A -** (Upper) Estimated prevalence of solid fuel heat sources by LSOA, England and Wales. (Lower) Estimated prevalence of solid fuel heat sources by LSOA, London. **B -** (Upper) Estimated concentration of solid fuel heat sources per km2 by LSOA, England and Wales. (Lower) Estimated concentration of solid fuel heat sources per km2 by LSOA, London. Estimated concentration values were truncated at the 5th and 95th percentiles for mapping.

Temporal trends in prevalence of wood burners in EPCs, by property type and region

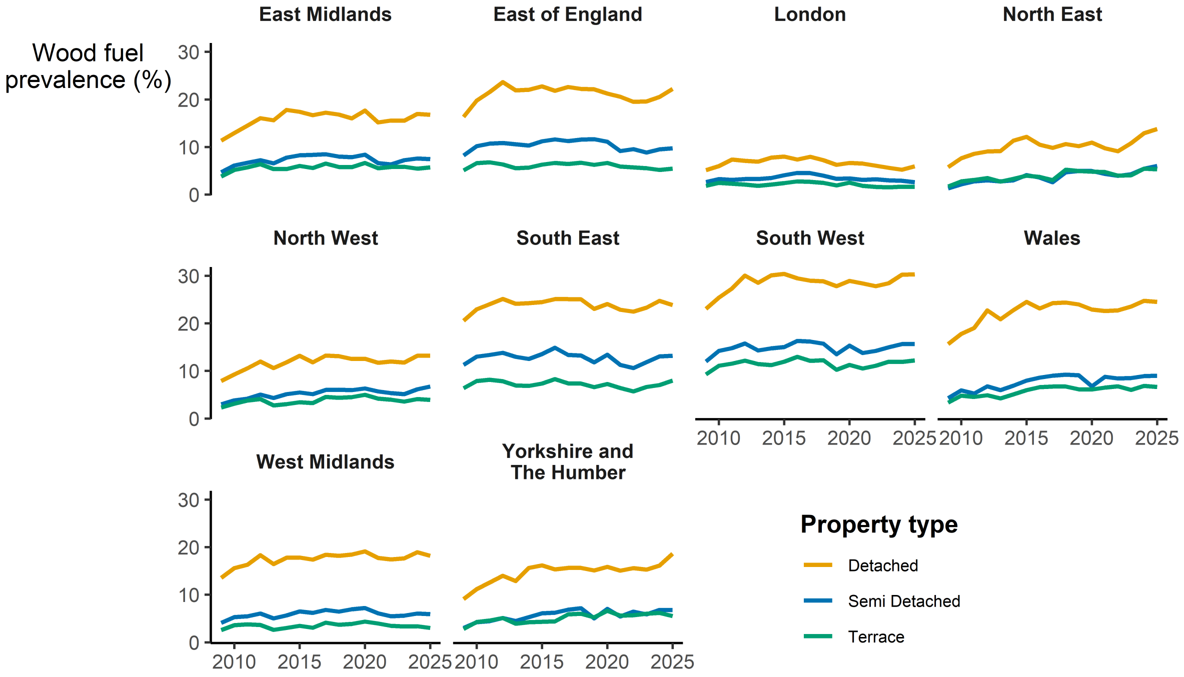

Figure 4: Prevalence of wood fuel heat sources in new EPC certificates from 2009-2025 by property type and region. For each property type, region, and year, we calculated the percentage of new EPCs which indicated the presence of a wood fuel heat source. We restricted the analysis to the sub-sample of detached, semi detached, and terraced houses, excluding flats and other accommodation types. We used all EPCs available in the dataset, including multiple EPCs for the same property.

Comparison of EPC concentration measure against LAEI gridded emissions

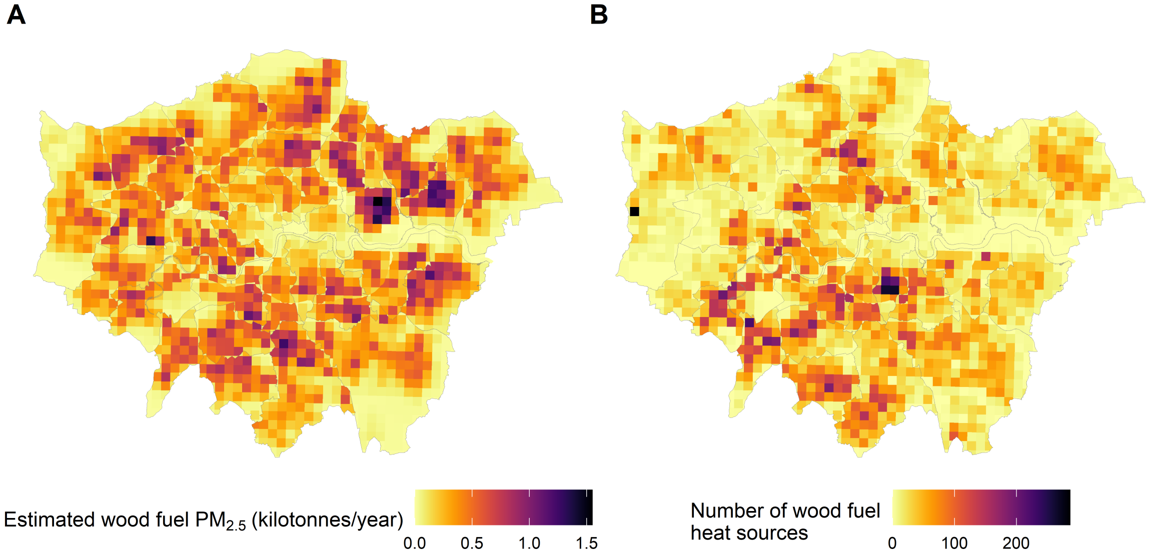

Figure 5: **A:** Estimated wood burning-derived $PM_{2.5}$ emissions in Greater London by 1km x 1km grid square using LAEI methodology (tonnes/year). **B:** Estimated number of wood burners by 1km x 1km grid square, using EPCs. We used OS AddressBase data to estimate the number of residential properties by property type in each 1km x 1km grid square, and used EPCs to estimate the percentage of properties with a wood fuel heat source by grid square and property type. We estimated the total number of wood burners by grid square by taking the product of the estimated number of properties and wood fuel prevalence by property type, before summing across property types.

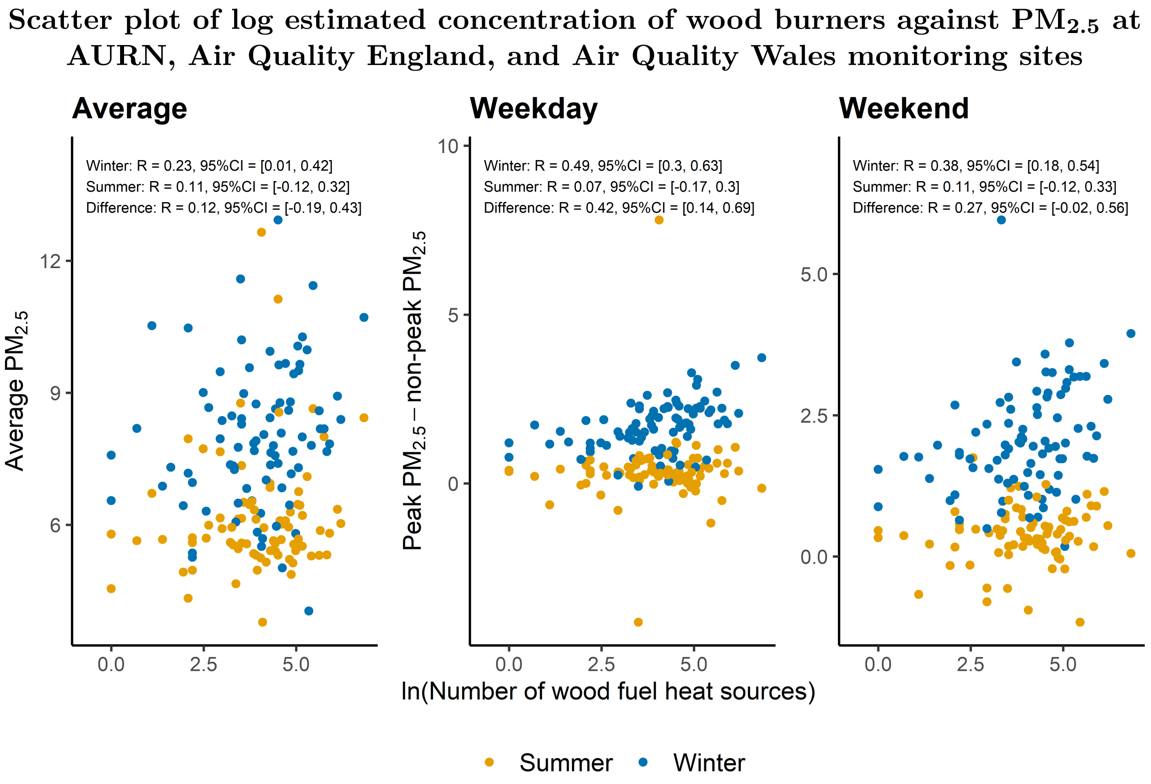

Figure 6: Left: Scatter plot of log estimated wood burner concentration against mean $PM_{2.5}$ at monitoring sites. Centre: Scatter plot of log estimated wood burner concentration against difference in weekday mean $PM_{2.5}$ measurements at peak vs. non-peak burning times. Right: Scatter plot of log estimated wood burner concentration against difference in weekend mean $PM_{2.5}$ measurements at peak vs. non-peak burning times. Peak burning times were defined as 7pm-1am, and non-peak times as 5am-5pm. Weekends were defined as Saturday and Sunday. The concentration of wood burners was estimated as the number of properties with a wood burner in the EPC data within a 1km circular radius of each monitoring site. We used sites classified as 'Urban Background' from the AURN, Air Quality England, and Air Quality Wales monitoring sites. The scatter plots are stratified into summer and winter observations. Each chart reports the central estimate and 95% confidence interval for the Spearman correlation coefficient (ρ). We calculated the 95% confidence intervals using the BCA bootstrap method with 10,000 replications. We also report bootstrap 95% confidence intervals for the difference between the summer and winter correlation coefficients.

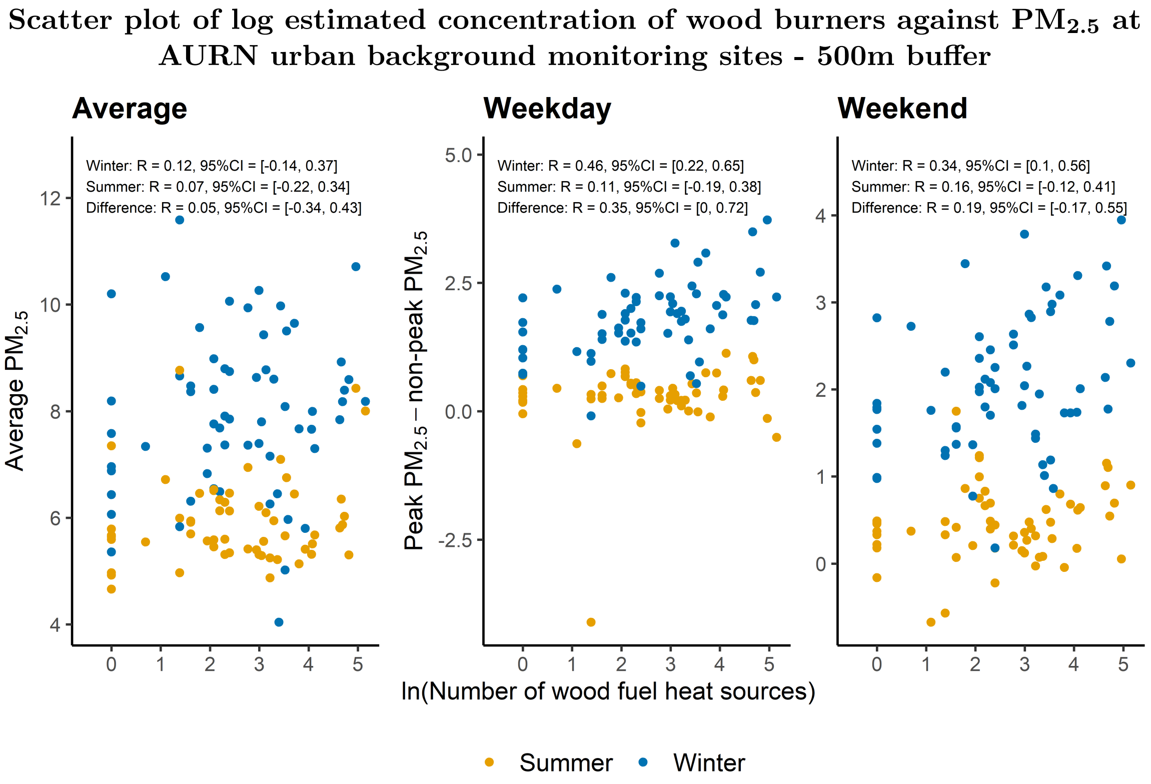

Figure 7: Left: Scatter plot of log estimated wood burner concentration against mean $PM_{2.5}$ at monitoring sites. Centre: Scatter plot of log estimated wood burner concentration against difference in weekday mean $PM_{2.5}$ measurements at peak vs. non-peak burning times. Right: Scatter plot of log estimated wood burner concentration against difference in weekend mean $PM_{2.5}$ measurements at peak vs. non-peak burning times. Peak burning times were defined as 7pm-1am, and non-peak times as 5am-5pm. Weekends were defined as Saturday and Sunday. The concentration of wood burners was estimated as the number of properties with a wood burner in the EPC data within a 1km circular radius of each monitoring site. We used sites classified as 'Urban Background' from the AURN. The scatter plots are stratified into summer and winter observations. Each chart reports the central estimate and 95% confidence interval for the Spearman correlation coefficient (ρ). We calculated the 95% confidence intervals using the BCA bootstrap method with 10,000 replications. We also report bootstrap 95% confidence intervals for the difference between the summer and winter correlation coefficients.

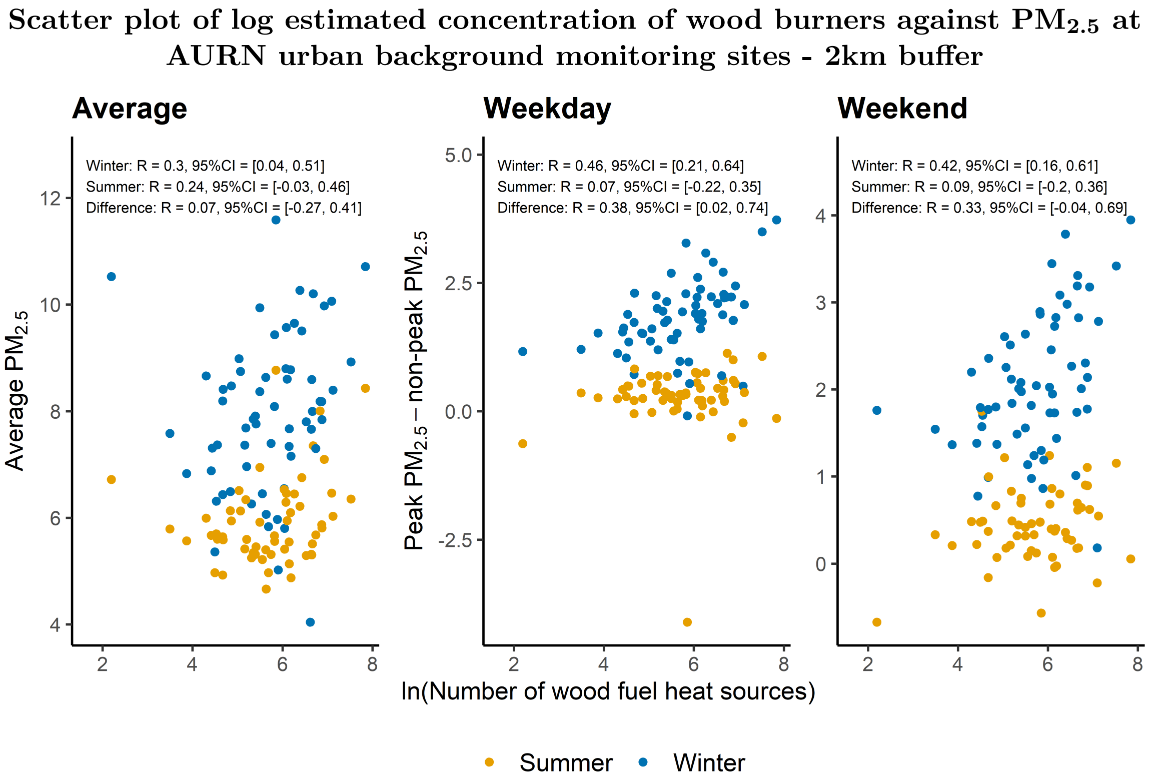

Figure 8: Left: Scatter plot of log estimated wood burner concentration against mean $PM_{2.5}$ at monitoring sites. Centre: Scatter plot of log estimated wood burner concentration against difference in weekday mean $PM_{2.5}$ measurements at peak vs. non-peak burning times. Right: Scatter plot of log estimated wood burner concentration against difference in weekend mean $PM_{2.5}$ measurements at peak vs. non-peak burning times. Peak burning times were defined as 7pm-1am, and non-peak times as 5am-5pm. Weekends were defined as Saturday and Sunday. The concentration of wood burners was estimated as the number of properties with a wood burner in the EPC data within a 1km circular radius of each monitoring site. We used sites classified as 'Urban Background' from the AURN. The scatter plots are stratified into summer and winter observations. Each chart reports the central estimate and 95% confidence interval for the Spearman correlation coefficient (ρ). We calculated the 95% confidence intervals using the BCA bootstrap method with 10,000 replications. We also report bootstrap 95% confidence intervals for the difference between the summer and winter correlation coefficients.

Scatter plot of log estimated housing density against $PM_{2.5}$ at AURN monitoring sites

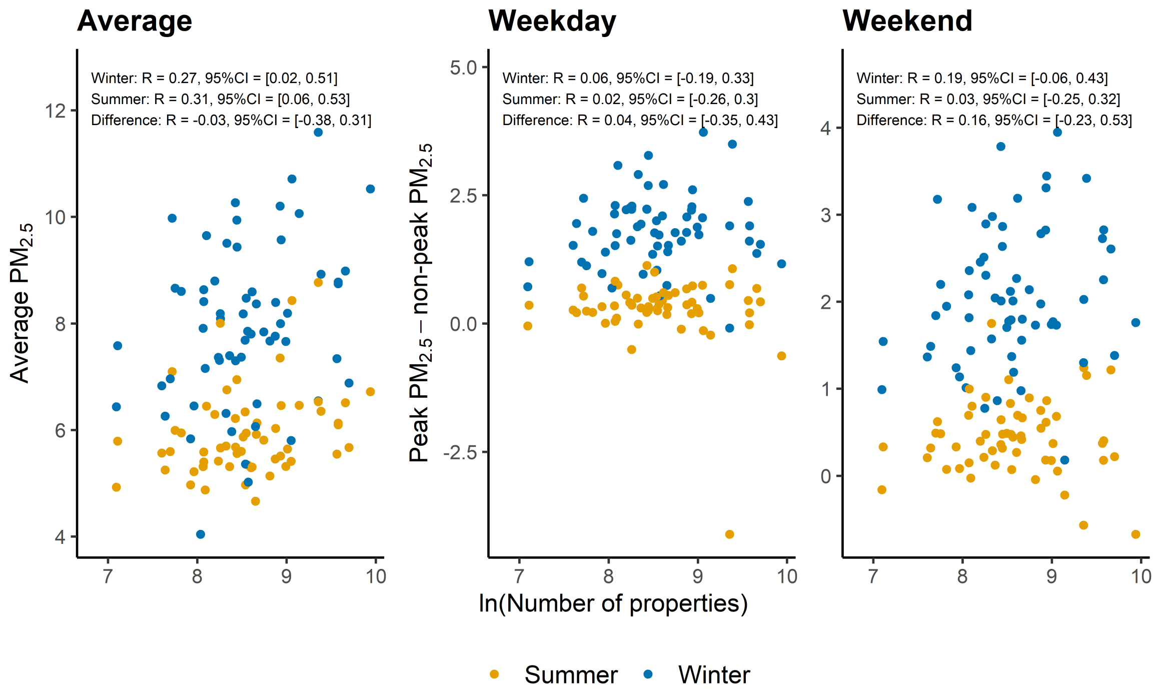

Figure 9: Left: Scatter plot of log estimated housing density against mean $PM_{2.5}$ at monitoring sites. Centre: Scatter plot of log estimated housing density against difference in weekday mean $PM_{2.5}$ measurements at peak vs. non-peak burning times. Right: Scatter plot of log estimated housing density against difference in weekend mean $PM_{2.5}$ measurements at peak vs. non-peak burning times. Peak burning times were defined as 7pm-1am, and non-peak times as 5am-5pm. Weekends were defined as Saturday and Sunday. Housing density was estimated as the number of properties in the EPC data within a 1km circular radius of each monitoring site. We used all AURN monitoring sites classified as 'Urban Background'. The scatter plots are stratified into summer and winter observations. Each chart reports the central estimate and 95% confidence interval for the Spearman correlation coefficient (ρ). We calculated the 95% confidence intervals using the BCA bootstrap method with 10,000 replications. We also report bootstrap 95% confidence intervals for the difference between the summer and winter correlation coefficients.

Scatter plot of log estimated concentration of wood burners against $PM_{2.5}$ at AURN monitoring sites - using NAEI grid squares

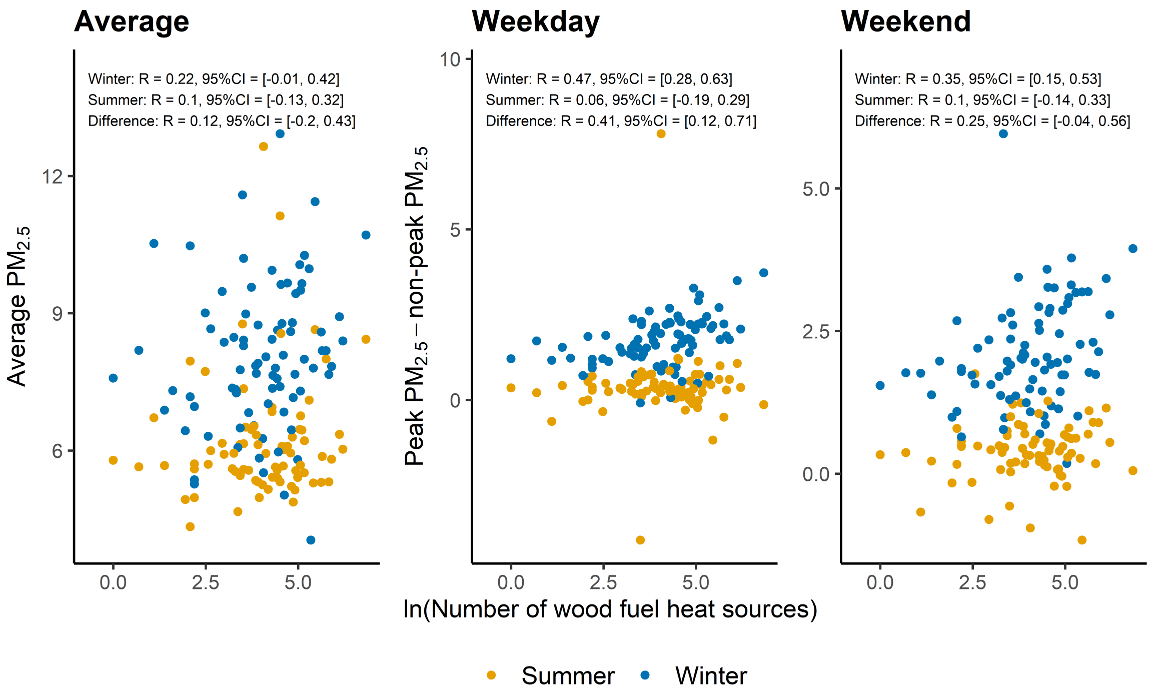

Figure 10: Left: Scatter plot of log estimated wood burner concentration against mean $PM_{2.5}$ at monitoring sites. Centre: Scatter plot of log estimated wood burner concentration against difference in weekday mean $PM_{2.5}$ measurements at peak vs. non-peak burning times. Right: Scatter plot of log estimated wood burner concentration against difference in weekend mean $PM_{2.5}$ measurements at peak vs. non-peak burning times. Peak burning times were defined as 7pm-1am, and non-peak times as 5am-5pm. Weekends were defined as Saturday and Sunday. The concentration of wood burners was estimated as the number of properties with a wood burner in the 1km by 1km grid square containing each monitoring site. We used all AURN monitoring sites classified as 'Urban Background'. The scatter plots are stratified into summer and winter observations. Each chart reports the central estimate and 95% confidence interval for the Spearman correlation coefficient (ρ). We calculated the 95% confidence intervals using the BCA bootstrap method with 10,000 replications. We also report bootstrap 95% confidence intervals for the difference between the summer and winter correlation coefficients.

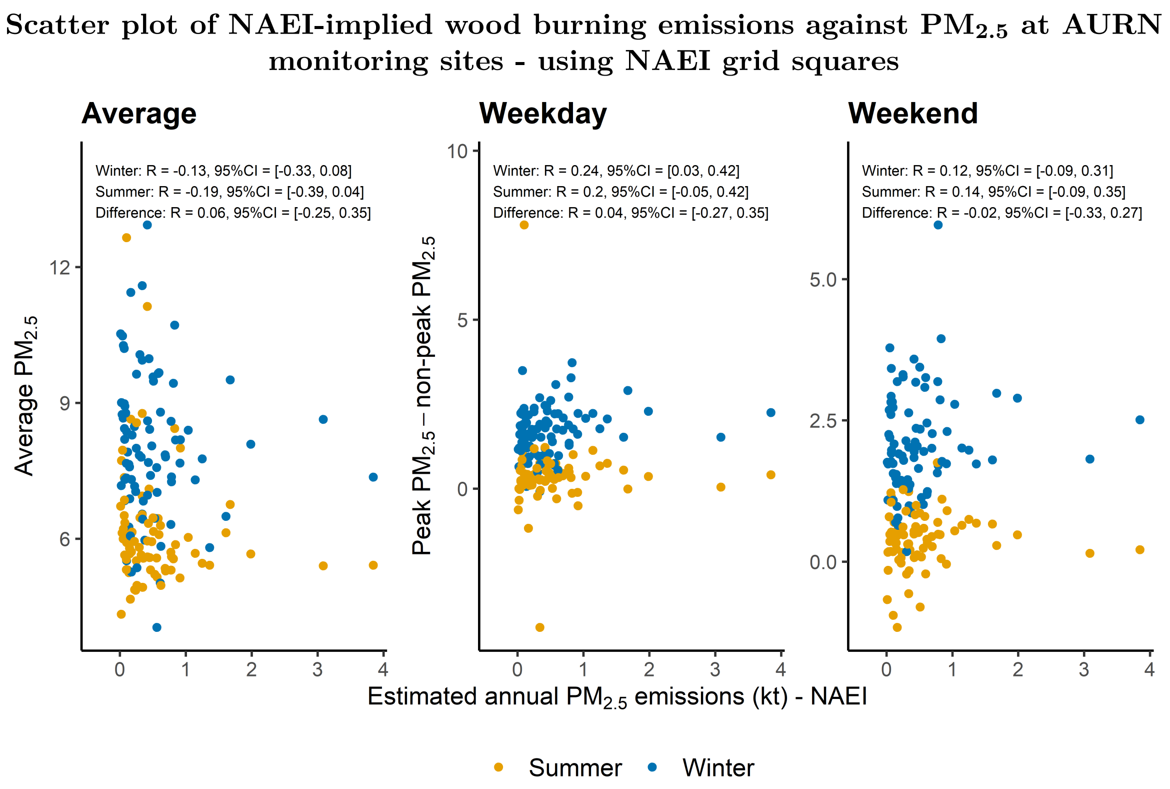

Figure 11: Left: Scatter plot of NAEI-estimated wood burning emissions against mean $PM_{2.5}$ at monitoring sites. Centre: Scatter plot of NAEI-estimated wood burning emissions against difference in weekday mean $PM_{2.5}$ measurements at peak vs. non-peak burning times. Right: Scatter plot of NAEI-estimated wood burning emissions against difference in weekend mean $PM_{2.5}$ measurements at peak vs. non-peak burning times. Peak burning times were defined as 7pm-1am, and non-peak times as 5am-5pm. Weekends were defined as Saturday and Sunday. The NAEI wood burning emissions estimates were reproduced from grid square data downloaded from the NAEI. We used all AURN monitoring sites classified as 'Urban Background'. The scatter plots are stratified into summer and winter observations. Each chart reports the central estimate and 95% confidence interval for the Spearman correlation coefficient (ρ). We calculated the 95% confidence intervals using the BCA bootstrap method with 10,000 replications. We also report bootstrap 95% confidence intervals for the difference between the summer and winter correlation coefficients.

Association between social deprivation, ethnicity, and prevalence of wood burners by region

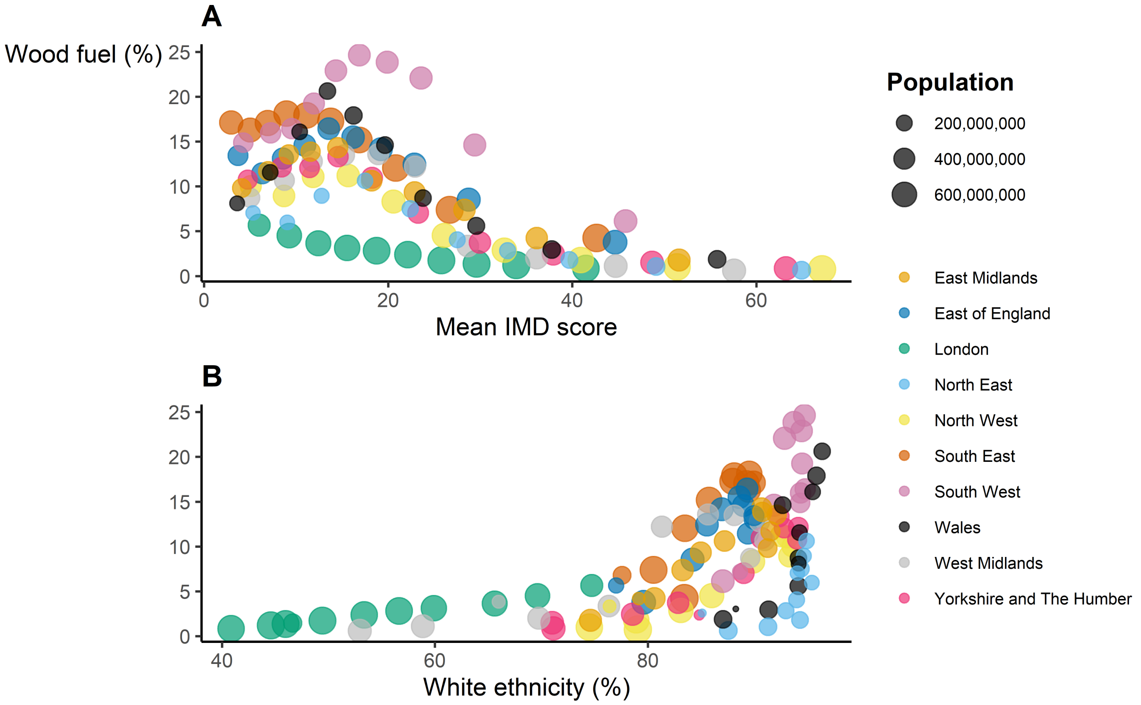

Figure 12: **A -** Association between mean IMD score and prevalence of wood fuel heat sources. Within regions, we grouped LSOAs by IMD decile and calculated the mean IMD score and prevalence of wood fuel heat sources for each region-IMD decile pair. Each point on the plot represents the mean IMD score and wood fuel prevalence for LSOAs within a given region and IMD decile. The size of the point corresponds to the total population within that region and IMD decile. **B -** Association between percentage of people from a white ethnic background and prevalence of wood fuel heat sources. We followed the same method as in **A** to generate the plot.

Association between social deprivation and prevalence of wood burners, by LSOA and urban/rural classification

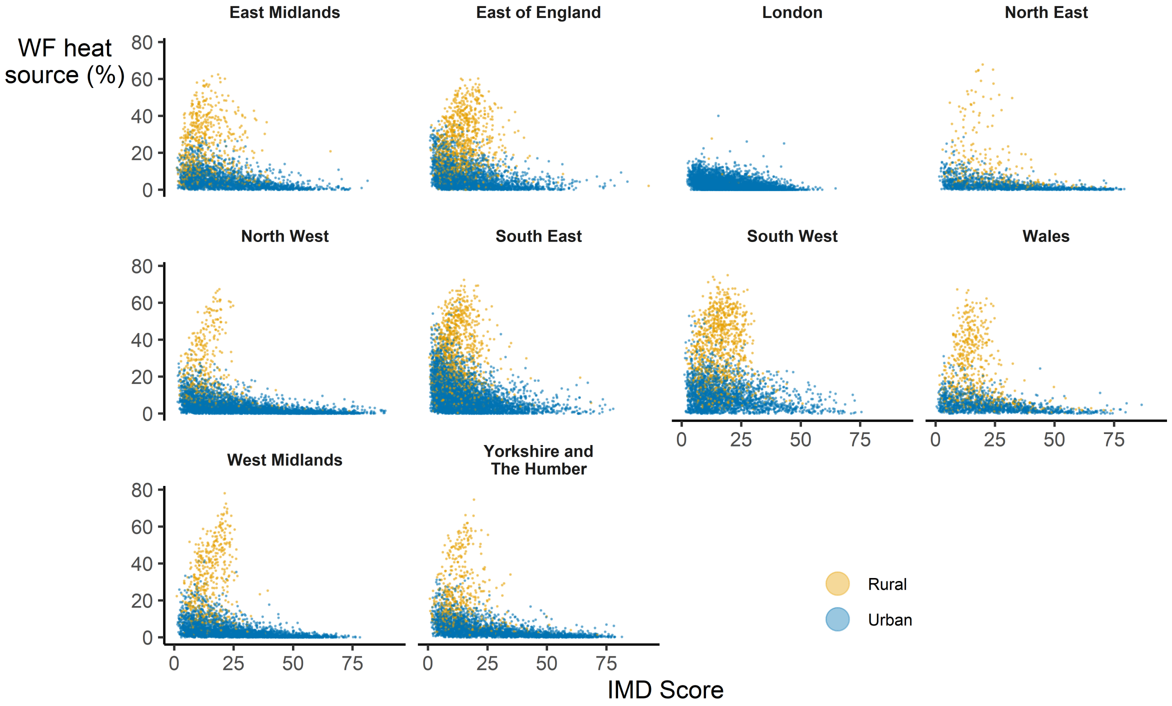

Figure 13: Scatter plot of wood fuel prevalence against IMD score by region. Each point represents an LSOA.

Association between ethnicity and prevalence of wood burners, by LSOA and urban/rural classification

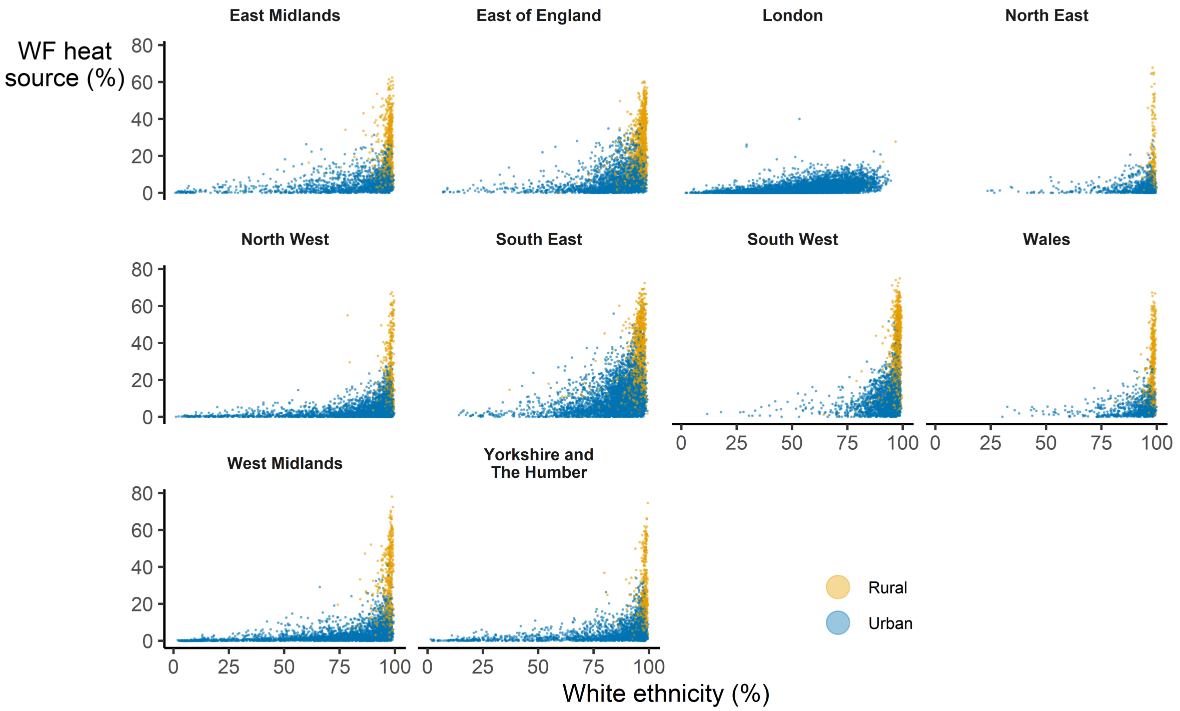

Figure 14: Scatter plot of wood fuel prevalence against percentage of people from a white ethnic background by region. Each point represents an LSOA.

Association between age and prevalence of wood burners, by LSOA and urban/rural classification

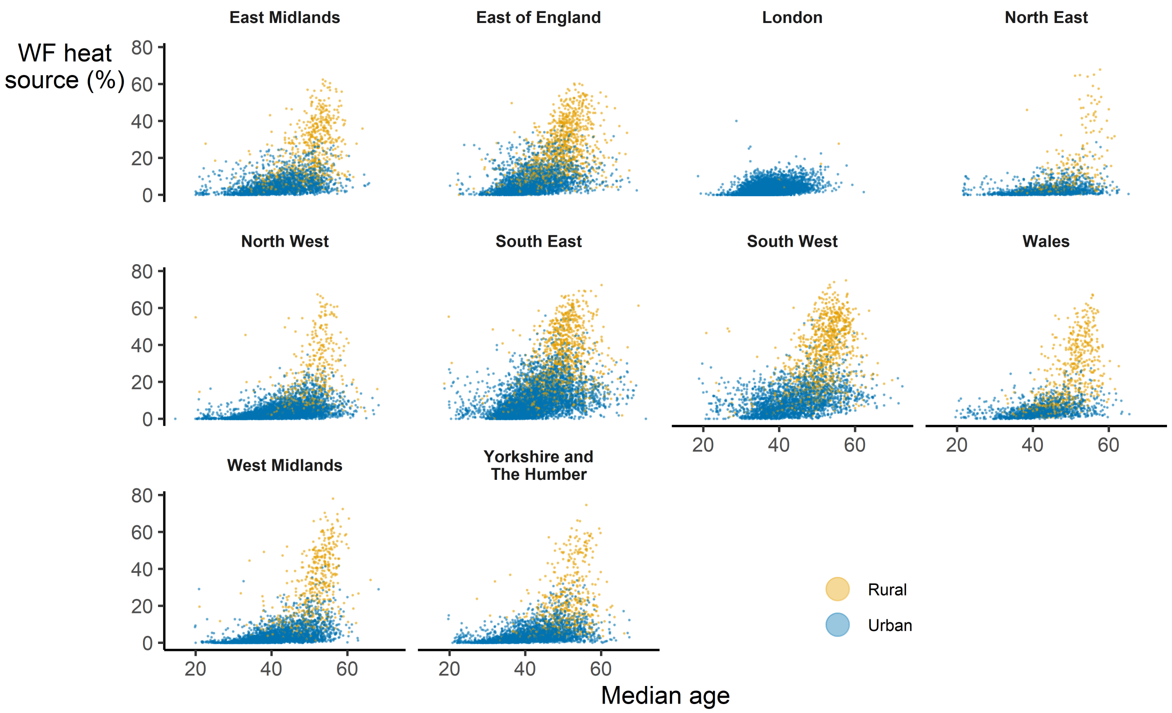

Figure 15: Scatter plot of wood fuel prevalence against median age by region. Each point represents an LSOA.

Tables

Table 1: Percentage of properties in the OS AddressBase and EPC dataset, by housing type

	OS AddressBase		EPC data	
Property type	N	Percentage	N	Percentage
Detached	5,830,768	21.0%	3,498,984	19.1%
Semi Detached	7,430,980	26.8%	4,590,086	25.0%
Terrace	7,603,717	27.4%	4,997,789	27.2%
Flat	6,606,064	23.8%	5,188,384	28.3%
Other accommodation	307,016	1.1%	9,652	0.1%

Note: Property types in the OS AddressBase and EPC data were re-categorised to match Census 2021 categories. In total, the OS AddressBase contained 28,638,819 properties. This table used data on EPCs up to October 2024.

Table 2: Property characteristics by presence of solid fuel heat source in England and Wales

	Solid fuel heat source	
	No (N = 17,122,175)	Yes (N = 1,586,345)
**Property type**		
Detached	2,825,767 (17%)	744,344 (47%)
Flat	5,294,287 (31%)	41,269 (2.6%)
House Form Missing	53,890 (0.3%)	2,782 (0.2%)
Other accommodation	9,744 ($<$ 0.1%)	243 ($<$ 0.1%)
Semi Detached	4,222,771 (25%)	449,583 (28%)
Terrace	4,715,716 (28%)	348,124 (22%)
**Tenure**		
newbuild	737,800 (4.7%)	18,718 (1.1%)
owner-occupied	8,539,885 (54%)	1,273,459 (84%)
rented (private)	3,267,043 (21%)	198,299 (13%)
rented (social)	3,130,467 (20%)	31,654 (2.1%)
Missing	1,446,980	64,215
**Smoke Control Area**		
No	8,294,583 (48%)	1,292,008 (81%)
Yes	8,827,592 (52%)	294,337 (19%)
**Urban**		
No	2,377,637 (15%)	823,921 (53%)
Yes	13,956,774 (85%)	731,085 (47%)
Missing	787,764	31,339

Note: We used the subset of unique properties in the EPC dataset. We excluded properties which had missing information on solid fuel presence. Each column shows the count of properties in the EPC database with the specified characteristic and solid fuel status, and the percentage of all properties of that solid fuel status with the specified characteristic. Missing values within characteristics are reported.

Table 3: Percentage of EPCs with a wood burner by property type and EPC number

Property type	First EPC	Second EPC	Third EPC	Fourth EPC
Number of EPCs: 2				
Detached	696,268 (19.4%)	691,327 (24.3%)		
Semi Detached	1,077,536 (7.2%)	1,070,212 (9.8%)		
Terrace	1,462,561 (4.8%)	1,454,760 (6.1%)		
Number of EPCs: 3				
Detached	104,145 (22.2%)	103,135 (24.8%)	103,022 (28.4%)	
Semi Detached	191,743 (6.6%)	191,687 (8.0%)	189,968 (9.4%)	
Terrace	277,958 (3.7%)	273,437 (4.4%)	275,301 (4.9%)	
Number of EPCs: 4				
Detached	16,250 (24.8%)	16,112 (26.2%)	16,002 (28.2%)	16,028 (31.9%)
Semi Detached	34,642 (6.3%)	34,579 (7.0%)	34,605 (7.8%)	34,404 (8.6%)
Terrace	54,275 (3.0%)	53,451 (3.3%)	53,446 (3.4%)	53,616 (3.8%)

Note: This table shows the percentage of EPCs with a wood burner by property type and EPC number, among the properties in the EPC data which had more than one EPC since 2009. We restrict our analysis to houses (excluding flats, other accommodation, and properties with missing house form) and restrict our analysis to properties with fewer than five EPCs, due to small sample size for higher numbers of EPCs. The total number of properties within rows are not equal due to changes in property classifications in the EPC dataset.

Table 4: Beta regression of prevalence of wood burners on LSOA-level characteristics

Characteristic	Coefficient	95% confidence interval
Urban	-0.951	(-0.971, -0.932)
Smoke Control Area	-0.275	(-0.299, -0.252)
Median age	0.039	(0.038, 0.041)
Percentage white ethnicity	0.008	(0.007, 0.009)
IMD score	-0.012	(-0.013, -0.011)
Region:		
East of England	0.061	(0.024, 0.097)
London	-0.117	(-0.164, -0.070)
North East	-0.439	(-0.489, -0.389)
North West	-0.087	(-0.124, -0.050)
South East	0.340	(0.306, 0.374)
South West	0.344	(0.309, 0.380)
Wales	-0.130	(-0.173, -0.087)
West Midlands	0.036	(-0.002, 0.074)
Yorkshire and the Humber	0.003	(-0.035, 0.042)

Note: This table displays results from a beta regression of LSOA-level prevalence of wood burners on geographic and socio-economic factors. The coefficients in the second column represent the the association between a one unit change in the independent variable and the change in the logit of the expected value of the (beta-distributed) outcome variable. Coefficients on region indicators are defined relative to the East Midlands. N = 35,504.

Table 5: Beta regression of prevalence of wood burners on LSOA-level characteristics - urban LSOAs

Characteristic	Coefficient	95% confidence interval
Smoke Control Area	-0.273	(-0.296, -0.250)
Median age	0.023	(0.021, 0.024)
Percentage white ethnicity	0.010	(0.009, 0.011)
IMD score	-0.018	(-0.019, -0.018)
Region:		
East of England	-0.036	(-0.078, 0.006)
London	-0.241	(-0.287, -0.196)
North East	-0.467	(-0.522, -0.412)
North West	-0.162	(-0.201, -0.123)
South East	0.266	(0.228, 0.303)
South West	0.213	(0.171, 0.254)
Wales	-0.285	(-0.337, -0.233)
West Midlands	-0.140	(-0.183, -0.098)
Yorkshire and the Humber	-0.023	(-0.069, 0.015)

Note: This table displays results from a beta regression of LSOA-level prevalence of wood burners on geographic and socio-economic factors among the subset of urban LSOAs. The coefficients in the second column represent the the association between a one unit change in the independent variable and the change in the logit of the expected value of the (beta-distributed) outcome variable. Coefficients on region indicators are defined relative to the East Midlands. N = 28,365.

Table 6: Beta regression of prevalence of wood burners on LSOA-level characteristics - rural LSOAs

Characteristic	Coefficient	95% confidence interval
Smoke Control Area	-0.543	(-0.625, -0.462)
Median age	0.062	(0.059, 0.066)
Percentage white ethnicity	0.008	(0.001, 0.015)
IMD score	-0.001	(-0.004, 0.001)
Region:		
East of England	0.124	(0.052, 0.196)
London	0.401	(-0.141, 0.943)
North East	-0.418	(-0.530, -0.306)
North West	-0.048	(-0.138, 0.041)
South East	0.409	(0.339, 0.479)
South West	0.395	(0.325, 0.466)
Wales	-0.028	(-0.111, 0.054)
West Midlands	0.257	(0.175, 0.339)
Yorkshire and the Humber	-0.003	(-0.088, 0.082)

Note: This table displays results from a beta regression of LSOA-level prevalence of wood burners on geographic and socio-economic factors among the subset of rural LSOAs. The coefficients in the second column represent the the association between a one unit change in the independent variable and the change in the logit of the expected value of the (beta-distributed) outcome variable. Coefficients on region indicators are defined relative to the East Midlands. N = 7,307.

A Data cleaning and linkage

We downloaded all 27.5 million EPCs from January 2009 to February 2025. We used UPRNs to link EPCs to active property records in the OS AddressBase data. We removed 327,639 (1%) duplicate EPCs for properties with available UPRNs.

We mapped UPRNs from EPCs to their respective geographies using the National Statistics UPRN Lookup for Great Britain as of May 2022. Of the non-duplicated records in the EPC data, 26 million records could be directly linked to a UPRN. We removed around 500,000 EPCs for properties without UPRNs, because it was not possible to determine whether these EPCs were the most recent EPC for the property. Next, we linked retained records to LSOA-level data including IMD score, ethnicity, median age, and rural-urban status.

The final EPC dataset contained 26 million records, among which there were 18.7 million unique properties.

B Search terms for primary and secondary heat source

Using the main EPC dataset, we generated indicator variables for whether a property's primary or secondary heat source was a solid fuel or wood fuel heat source using keyword searches.

At the time of analysis, the primary heat description contained 1,164 distinct entries, and the secondary heat description included 93 entries. The most common primary heat description was 'boiler and radiator, mains gas', with 20.7 million entries. The next most common descriptions were 'electric storage heaters' (1.7 million entries) and 'room heaters, electric' (1.3 million entries). The most common secondary heat descriptions were 'none' (16.2 million entries), 'room heaters, mains gas' (4.4 million entries), and 'room heaters, electric' (3.4 million entries).

From the primary and secondary heat source data fields of EPCs, we identified entries containing references to solid fuels and wood fuels in English and Welsh. The search terms used to identify solid fuels were 'wood', 'coal', 'anthracite', 'mineral', 'smokeless fuel' and 'dual fuel', and their Welsh language equivalents. The search terms used to identify wood fuels were 'wood' and 'dual fuel'.

C Technical appendix

This section provides further details on the method used to derive the estimated concentration and prevalence of wood fuel heat sources.

Let R denote the number of geographical regions, and let $R_{ir} \in \{0,1\}$, $i = 1, ..., N$, $r = 1, ...R$ denote the binary indicator that property i belongs to region r. $N_r = \sum_{i=1}^{N} R_{ir}$ is the number of properties in region r.

Consider a random variable Y defined as follows:

$$Y_i = \begin{cases} 1 & \text{if property i has a wood fuel heat source} \\ 0 & \text{otherwise} \end{cases} \tag{1}$$

Our estimand of interest is the prevalence of wood fuel heat sources by geographic region, π_r:

$$\pi_r = E(Y_i | R_{ir} = 1) \tag{2}$$

Let $S_i \in \{0,1\}$ denote a binary indicator for whether property i is observed in the EPC data. Then, $N_{rs} = \sum_{i:S_i=1}^{N} R_{ir}$ is the number of properties in region r observed in the EPC data. The simple within-region sample mean is defined as:

$$\bar{\pi}_r = \frac{1}{N_{rs}} \sum_{i:R_{ir}=1,\, S_i=1}^{N} Y_i \tag{3}$$

Assuming that Y_i has finite variance, this estimator is consistent for the expected prevalence of wood fuel heat sources by region, conditional on being observed in the EPC data:

$$\bar{\pi}_r \xrightarrow{p} E(Y_i | R_{ir} = 1, S_i = 1) \tag{4}$$

In general, this quantity will only equal the expectation in Equation 1 if there is no selection bias within regions (i.e. $Y_i \perp\!\!\!\perp S_i | R$).

We calculate an alternative prevalence measure using Census 2021 information on the share of housing types by geographic region. Let $H_{ih} \in \{0,1\}$, $i = 1, ..., N$, $h = 1, ...H$ denote whether property i belongs to housing type h. $N_{rh} = \sum_{i:R_{ir}=1}^{N} H_{ih}$ is the number of properties of housing type h in region r, and $\frac{N_{rh}}{N_r}$ is the proportion of housing type h in region r. $N_{rhs} = \sum_{i:R_{ir}=1,S_i=1}^{N} H_{ih}$ is defined as the number of households of housing type h in region r observed in the EPC data.

First, we estimate the prevalence of wood fuel heat sources within household types and LSOAs from the EPC data:

$$\hat{\pi}_{rh} = \frac{1}{N_{rhs}} \sum_{i:\{R_{ir},H_{ih},S_i\}=1}^{N} Y_i \tag{5}$$

Then, we calculate the estimated prevalence by LSOA, weighted by the Census-derived proportion of housing types by LSOA:

$$\hat{\pi}_r = \frac{1}{N_r} \sum_{h=1}^{H} \hat{\pi}_{rh} N_{rh} \tag{6}$$

This method relies on the relatively weaker assumption that within regions and housing types, selection is uncorrelated with the probability of having a wood fuel heat source:

$$Y_i \perp\!\!\!\perp S_i | R, H$$

To generalise Equation 6 to higher-level geographies, we construct similar estimates by averaging LSOA-level prevalence measures, weighted by the total number of properties within the LSOA.

We take a similar approach to predict the concentration of wood fuel heat sources by region, except that we take a weighted sum instead of a weighted average in Equation 6, before dividing by the area of the LSOA in km^2.
